# How Obstacles Perturb Population Fronts and Alter Their Genetic Structure

**DOI:** 10.1371/journal.pcbi.1004615

**Published:** 2015-12-22

**Authors:** Wolfram Möbius, Andrew W. Murray, David R. Nelson

**Affiliations:** 1 Department of Physics, Harvard University, Cambridge, Massachusetts, United States of America; 2 FAS Center for Systems Biology, Harvard University, Cambridge, Massachusetts, United States of America; 3 Department of Molecular and Cellular Biology, Harvard University, Cambridge, Massachusetts, United States of America; University of Bern, SWITZERLAND

## Abstract

As populations spread into new territory, environmental heterogeneities can shape the population front and genetic composition. We focus here on the effects of an important building block of heterogeneous environments, isolated obstacles. With a combination of experiments, theory, and simulation, we show how isolated obstacles both create long-lived distortions of the front shape and amplify the effect of genetic drift. A system of bacteriophage T7 spreading on a spatially heterogeneous *Escherichia coli* lawn serves as an experimental model system to study population expansions. Using an inkjet printer, we create well-defined replicates of the lawn and quantitatively study the population expansion of phage T7. The transient perturbations of the population front found in the experiments are well described by a model in which the front moves with constant speed. Independent of the precise details of the expansion, we show that obstacles create a kink in the front that persists over large distances and is insensitive to the details of the obstacle’s shape. The small deviations between experimental findings and the predictions of the constant speed model can be understood with a more general reaction-diffusion model, which reduces to the constant speed model when the obstacle size is large compared to the front width. Using this framework, we demonstrate that frontier genotypes just grazing the side of an isolated obstacle increase in abundance, a phenomenon we call ‘geometry-enhanced genetic drift’, complementary to the founder effect associated with spatial bottlenecks. Bacterial range expansions around nutrient-poor barriers and stochastic simulations confirm this prediction. The effect of the obstacle on the genealogy of individuals at the front is characterized by simulations and rationalized using the constant speed model. Lastly, we consider the effect of two obstacles on front shape and genetic composition of the population illuminating the effects expected from complex environments with many obstacles.

## Author Summary

Geographical structure influences the dynamics of the expansion of populations into new habitats and the relative importance of the evolutionary forces of mutation, selection, genetic drift, and gene flow. While populations often spread and evolve in highly complex environments, simplified scenarios allow one to uncover the important factors determining a population front’s shape and a population’s genetic composition. Here, we follow this approach using a combination of experiments, theory, and simulations. Specifically, we use an inkjet printer to create well-defined bacterial patterns on which a population of bacteriophage expands and characterize the transient perturbations in the population front caused by individual obstacles. A theoretical understanding allows us to make predictions for more general obstacles than those investigated experimentally. We use stochastic simulations and experimental expansions of bacterial populations to show that the population front dynamics is closely linked to the fate of genotypes at different parts of the front. We anticipate that our findings will lead to understanding of how a wide class of environmental structures influences spreading populations and their genetic composition.

## Introduction

Populations expand into new territory on all length and time scales. Examples include the migration of humans out of Africa [1], the recent invasion of cane toads in Australia [2], and the growth of colonies of microbes. Although populations often persist long after invading [3], events during their spread can have long-lasting effects on their genetic diversity [4, 5]. Considerable effort has been undertaken to understand the role of the invasion process on the evolutionary path of the population: The small population size at the edge of the advancing population wave amplifies genetic drift, reducing genetic diversity, which can culminate in the formation of monoclonal regions [4]. The fate of mutations—deleterious, neutral, or beneficial—occurring in the course of the expansion depends on the location of their appearance with respect to the edge of the wave [4, 6–10]. While the genetic consequences of such range expansions have been studied in the field [11, 12], the complexity of natural populations makes it difficult to draw general conclusions. Laboratory expansions of microbes have thus become a useful tool to illustrate, test, and inspire theoretical predictions [13–16].

The majority of theoretical and experimental work on range expansions has focused on homogeneous environments while habitats in nature are often spatially heterogeneous with regard to dispersal or population growth, the two processes that lead to the expansion. Incorporating environmental heterogeneity into models of spreading populations [3, 4, 17, 18] raises complex problems. Heterogeneity can affect any parameter that controls population dispersal or growth and there can be many spatial patterns of heterogeneity. Ecologists and population geneticists often focus on different consequences of environmental heterogeneity. Work in population dynamics and ecology typically concentrates on the effect of heterogeneity on invasibility and the speed and impact of an invasion in such environments [3, 17, 19–22] and is closely linked to the mathematical and physical aspects of front propagation [23, 24]. In contrast, population genetics studies usually assume a successful invasion and ask how environmental heterogeneities affect the population’s genetic composition [4]. Although heterogeneous carrying capacities [25], fragmented environments [26], single corridors or obstacles [8, 27], and environmental patterns found on earth [7, 8, 28] have been addressed from a theoretical perspective, a systematic understanding is still missing. In this work, we study the population dynamics and relate the dynamics of the population front to the consequences on the genetic composition of the spreading population, thereby linking the evolutionary and ecological consequences of range expansions.

**Fig 1:**
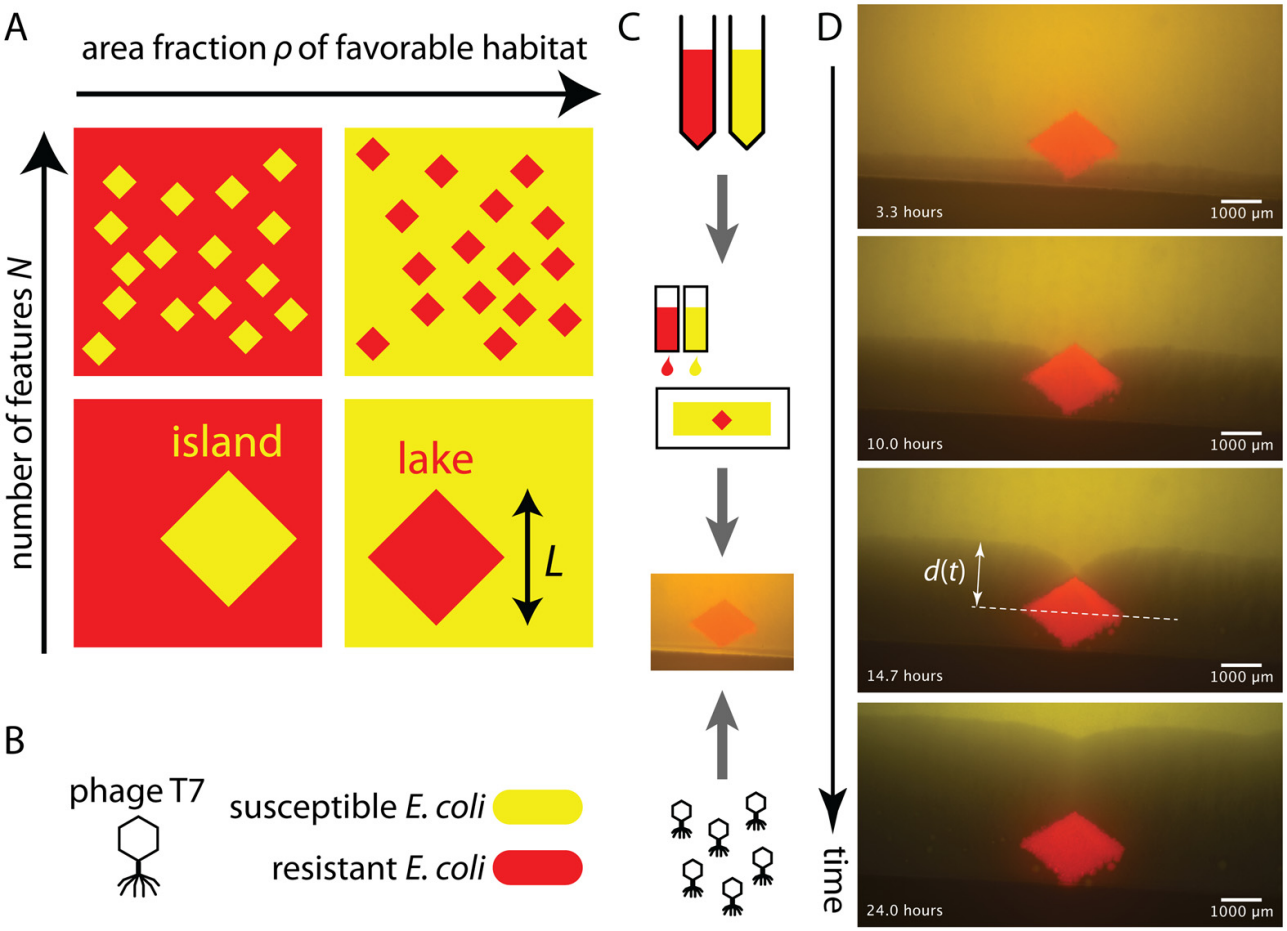
Heterogeneous environments and phage system to study effect of isolated obstacles. **(A)** Classification of environments composed of regions that permit or prohibit reproduction based on the area fraction of favorable habitat, the fraction of the habitat that allows growth, *ρ*, and number *N* of features of linear size *L*. The features are embedded in the environment accessible to the spreading population. In this work, we focus on the ‘lake scenario’, i.e., regions that prohibit growth (red) distributed in an environment that permits growth (yellow). **(B)** Bacteriophage as an experimental model for expansion in heterogeneous environments: for bacteriophage T7, a lawn of susceptible *E. coli* (wild-type, WT) represents an environment of good growth conditions (yellow fluorescent marker), while a region with resistant *E. coli* (*waaC*Δ, red fluorescent marker) represents poor growth conditions. **(C)** Schematic diagram of the assay to observe plaque growth in well-defined reproducible environments. A digital representation serves as input for printing bacterials strains, both wild-type and phage-resistant, on an agar patch using a consumer inkjet printer. After the pattern has grown, phage is added and plaque growth is observed. See Materials and Methods for details. **(D)** Snapshots of plaque propagation (dark regions) around a rhombus-shaped area of resistant bacteria (red) printed in a sea of sensitive bacteria (yellow). The plaque front remains flat until it reaches the widest part of the obstacle. There, it curves into a region roughly as wide as the obstacle. Once the front reaches the top of the obstacle, a kink forms, which then slowly heals. This panel also illustrates *d*(*t*), the distance the front has traveled beyond the obstacle at time *t*, where *d* = 0 at the point of maximal width of the obstacle. See S1 Video for the complete time lapse information.

We want to understand what happens when expanding populations confront environmental heterogeneities. For simplicity, assume that at each point the environment is a high quality habitat (large growth rate at population density well below carrying capacity) or a low quality habitat (very small or zero growth rate). What constitutes low quality habitats depends on the population: For a macroscopic expansion of a terrestrial animal or a plant, lakes and mountain ranges are examples of low quality habitat. For microbes, regions with poor nutrients may represent an obstacle to colony growth. If *ρ* is the fraction of the environment that allows growth, we can distinguish between two scenarios: For 0 < *ρ* ≪ 1/2, the ‘island scenario’, a largely inhospitable environment is interrupted by islands or oases of growth; in contrast, for 1/2 ≪ *ρ* < 1, the ‘lake scenario’, a largely hospitable environment is punctuated by obstacles that impede growth (Fig 1A). The island scenario, reminiscent of stepping stone models of population genetics [29], with a weak coupling between nearby islands by migration, is a situation where genetic drift can lead to genetic uniformity on individual islands due to founder effects [30]. Here, we address the lake scenario in the context of spatial expansions.

In addition to the fraction of the habitat not occupied by obstacles, *ρ*, we must also consider the number of obstacles, *N*, in the new habitat to be invaded. When *N* ≫ 1, i.e., when many (non-overlapping) obstacles are engulfed as the range expansion progresses, we expect that the principal effect of interest from a population dynamics perspective is the speed of the invasion and the roughness of the population front (top of Fig 1A). If, at the other extreme, the expansion domain only includes one obstacle with a size comparable to the size of the habitat invaded, the obstacle’s size, shape, and spatial arrangement are expected to greatly influence the shape of the front at the length scale of the habitat (bottom of Fig 1A).

We study the effect of isolated obstacles on the spread of populations. Using a combination of experiment, theory, and simulation, we characterize the obstacle’s effect in a regime of sizes where the shape of the front is well-described by a phenomenological model of expansion with constant speed. The constant speed model reveals general effects which hold independently of the mechanisms for population spread: The perturbation in the population front induced by the obstacle is determined by the obstacle’s width, but not by its precise shape. The front shape, induced by the obstacle, governs the effect on the genetic composition of the expanding population. Expanding past obstacles reduces genetic diversity and privileges genotypes that just miss an obstacle’s edges, an example of ‘geometry-enhanced genetic drift’, effects which are reflected in the genealogy of individuals at the front. In addition to the phenomenological model of front shape, we study a reaction-diffusion model, which enables us to compare experiments to a theoretical description in more detail and to understand the utility of the constant speed model in situations that extend beyond the experimental system studied here.

To derive these findings, we combine an analytical model, simulations, and experiments. While the experiments are the basis for theoretical work, they also allow us to test theoretical predictions. The analytical model provides the opportunity to make predictions for a variety of environments and length scales while simulations are used to explore regimes not accessible to analytical solutions. In addition to using established theoretical and experimental methods to study expanding populations, we present a new laboratory model system which allows us to quantitatively study population spread in heterogeneous environments: the expansion of bacterial viruses (bacteriophage) on a lawn of sensitive and resistant bacteria. Patches of resistant bacteria represent obstacles to the spread of the phage and can be generated using a printing technique, allowing us to quantitatively test predictions.

The growth dynamics of the phage system with phage and bacterial host differs from the one-species system with logistic growth, the FKPP equation [43], often used to study population expansions theoretically and used as the basis for our reaction-diffusion model. The dynamics of phage spread is governed by the density of phage, the density of bacteria, and the density of uninfected bacteria. However, at long times, the profiles of infected and uninfected bacteria are slaved to the profile of a traveling population wave of phage with a constant speed, and its motion mimics the dynamics of the simpler FKPP model. This similarity makes sense, because it is well-known that under broad conditions the solution to reaction-diffusion equations produces traveling waves with constant velocity whose speed is determined by linearization at the foot of the wave [43]. Hence, we expect that our phage system reflects well aspects of range expansions that depend on the biology at the leading edge of the front and believe that it offers the prospect of studying demographic and evolutionary processes in complex, yet well-defined environments.

## Results

### Transient perturbations of plaque boundary by obstacles

To explore the effects of obstacles on the population front dynamics, we employed a microbial model system, bacteriophage T7 spreading on a lawn of *E. coli* cells. Phage T7, a virus of *E. coli*, infects bacterial cells and lyses them, releasing a large number of new phage particles which undergo passive dispersal and can infect nearby cells, a cycle of growth and replication that leads to an advancing population front. Phage T7 must kill the bacteria it infects [31] and its spread on a bacterial lawn is revealed by the growth of plaques (clearings in the lawn due to lysis of bacteria), a fast process easily visualized by bright-field or fluorescence microscopy. We produce a heterogeneous environment for phage spread by incorporating regions which do not support propagation of the population wave: While a wild-type bacterial region (marked by a constitutively expressed yellow fluorescent protein) corresponds to a region supporting phage production, a resistant bacterial patch, an obstacle (similarly marked by a red fluorescent protein (mCherry)), does not, see Fig 1B and Materials and Methods. A lawn with regions of susceptible and resistant bacteria represents a static, heterogeneous environment that the phage population travels across during its expansion and that can be easily visualized.

We designed an assay that allowed linear fronts of expanding phage populations to encounter obstacles of defined shape. We modified a method that used a consumer inkjet printer to print sugar solutions [32] to deposit bacteria in defined patterns on agar surfaces (such as was done using custom-made equipment [33]). The printer produces a field of bacteria on a rectangular (3.5 × 2 cm^2^) agar patch at sub-mm resolution (Fig 1C, Materials and Methods, S1 Protocol and S1 Fig). The printed founder cells grow into a lawn, which is inoculated with a linear front of phage T7 close to or at the region with resistant bacteria (Fig 1D). The phage population spreads on this heterogeneous lawn, with repeated cycles of infection and lysis of the susceptible bacteria leading to the loss of fluorescence and the expanding dark region. Fig 1D shows such a printed pattern at different stages of the phage invasion (see also S1 Video): A linear population wave of phage encounters the region of resistant bacteria, the obstacle. The front curves as it passes the widest part of the obstacle and the two curved regions move along the far side of the object until they unite with each other, giving rise to a kink that disappears with time as the front moves beyond the obstacle.

**Fig 2:**
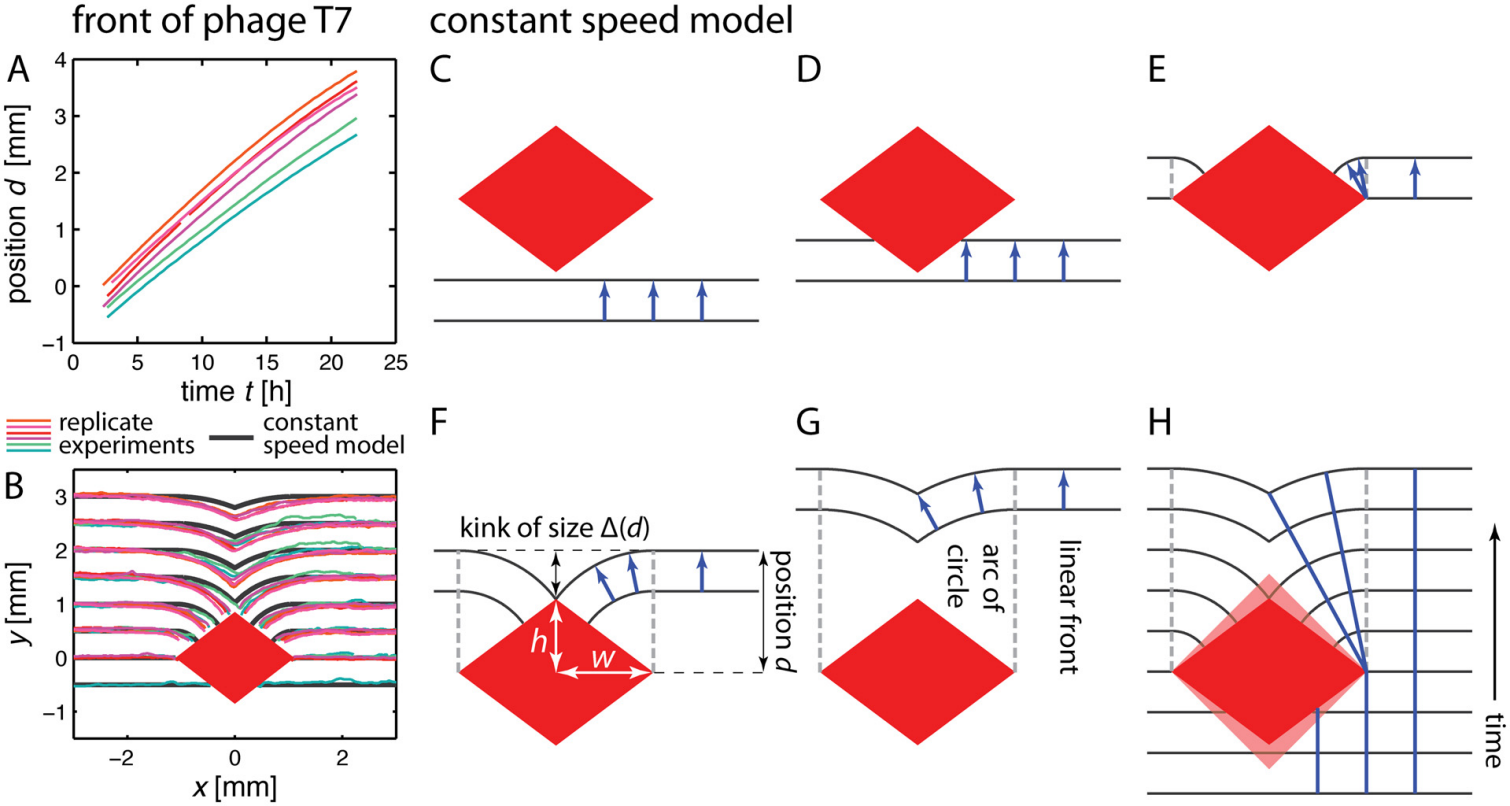
Position and shape of front of phage T7 and illustration of constant speed model. **(A)** Position of the front relative to the obstacle center (see Fig 1D) as inferred by quantitative image analysis (Materials and Methods), as a function of time. Front position is indicated as the mean of front position determined 3 mm to left and right of the obstacle center, respectively. Each color indicates one out of six replicates. **(B)** Front shape at different front positions *d* relative to the obstacle for six replicates together with shape of the obstacle as inferred by image analysis (Materials and Methods), colors corresponding to panel (A). The black line corresponds to the prediction by the constant speed model illustrated in the following panels: **(C-G)** Constructing the front shape encountering a rhombus-shaped obstacle using the constant speed model. Blue arrows have equal length and indicate the distance and direction of front movement over a small time interval. Panels C to E illustrate how the front is broken as it encounters the obstacle and then curves around on the far side of the obstacle. Panels F and G show how a kink of size Δ, which forms when the disrupted front reunites, heals downstream from the obstacle due to the radius of the circular region that defines the perturbed front increasing with position *d* as the front moves beyond the obstacle. **(H)** Superimposition of the fronts in panels C to G (compare black lines in panel (B) and Fig 3A and 3C) with blue lines indicating the path of a virtual marker at the front. For a lightly shaded rhombus with same width, but larger height, the shape of the front is unchanged (but the circular arcs are shorter and the kink forms later).

We used the difference between two consecutive images to detect the front of the plaque (Materials and Methods), and studied the front position as a function of time. We define the unperturbed front position *d*(*t*) as the position of the plaque’s edge at a horizontal distance of ±3 mm away from the obstacle center as displayed in Fig 1D. Fig 2A, displaying front position as a function of time, shows that the plaque grows at an approximately constant speed, but slows down slightly over time, presumably due to *E. coli* entering stationary phase [34]. The varying slope illustrates variation in front speed among replicates. Overall, the plaque extends with an approximately constant speed of 0.2 mm/h. The profile of the fluorescence signal in direction of the moving front is constant in time as shown in S2 Fig. Fig 2B shows the front shape over time for multiple replicates. The evolution of the front is very similar across replicates, despite small variations in front speed and initial conditions. While the perturbation of the front by the obstacle and the formation of a kink is intuitive at first, we aimed for a quantitative model which can describe front shape and make predictions which can be tested experimentally.

### Constant speed model

An arguably most minimal model assumes that the front moves with constant speed in direction normal to the front and ignores the microscopic details of how phage replicate inside bacteria and diffuse outside them. We dubbed this the ‘constant speed model’. Fig 2C–2G illustrates the dynamics of a front encountering a rhombus-shaped obstacle: (C) An initially linear front moves forward uniformly until the obstacle is encountered. (D) When it encounters the obstacle, the front stays linear, but is interrupted in the interval where it would overlap with the obstacle. (This is different from scenarios where a front of material encounters an obstacle and the obstacle “pushes” the material to the sides.) (E) Beyond the obstacle’s widest points, propagation with constant speed creates circular arcs in the shade of the obstacle that are connected to a linear front on either side of the obstacle. The circular elements span a region given by the obstacle’s width and encounter the obstacle at a 90° angle. (F) For a rhombus with height 2*h* and width 2*w*, the arcs from the two sides meet and a kink forms after the front has traveled a distance $\sqrt{w^{2}+h^{2}}$ beyond the point of maximum width. (G) The kink then heals due to the increasing radii of the circular segments, i.e., the size of the indent Δ decreases (Δ(*d*) ∼ 1/*d* for large “downstream” distances *d*, where *d* is the distance perpendicular to the front from the widest point of the obstacle to the unperturbed portion of the front, see below). Fig 2H shows that the height of the rhombus-shaped obstacle does not play a role in determining front shape and thus the size of the indent while the kink heals: For an obstacle which is taller (light red rhombus), the kink forms later and the circular arc where it forms is correspondingly shorter, but the shape of the downstream kink is independent of the obstacle height. Moreover, the circular shapes of the front on the far side of rhombus-shaped obstacles all fall onto the same master curve when plotted in units of *w*. A calculation shows that the indent size Δ as function of position *d* is indeed independent of *h* for rhombus-shaped obstacles and shows the same functional behavior if all lengths are expressed in units of *w* (see also S1 Appendix):

$$\begin{array}{ccc} \frac{\Delta (d)}{w} & = & \frac{d}{w}\left(1-\sqrt{1-\frac{w^{2}}{d^{2}}}\right)\;\underset{d\gg w}{\approx }\;\frac{w}{2d}\;. \end{array}$$ (1)

Counterintuitively, the width of the obstacle thus is a more important predictor of downstream front shape than obstacle height. For rhombus-shaped obstacles, obstacle height determines where the kink forms, but not the shape of the front after formation of the kink. Below, we will discuss more general obstacle shapes and the influence of obstacle size on the applicability of the constant speed model.

### Comparing the constant speed model to front propagation in printed environments

The constant speed model predicts the shape of the front at a given front position relative to the obstacle, this way allowing direct comparison with the experimentally determined front shapes in Fig 2B. While the constant speed model captures overall front shape and the transient character of the perturbation, the details of the predicted front (black line) differ from the experimental data (colored lines). The experimental profiles consistently lag behind the predicted front.

**Fig 3:**
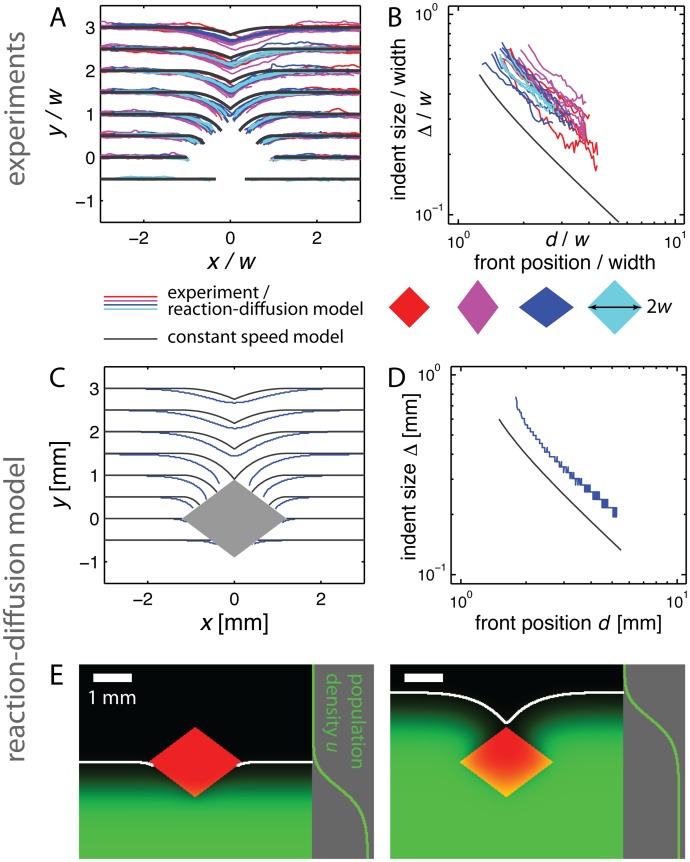
Comparison of phage fronts and reaction-diffusion model to constant speed model predictions. **(A)** Experimentally determined plaque front shapes (*x*, *y*) for different obstacles with length in units of *w*, half the obstacle width. Colored rhombuses indicate the ratio of height to width in the four cases (height and width are about 1.2 mm and 0.9mm, see Materials and Methods) and correspond to the color of the front shapes depicted. While the data collapse as predicted by the constant speed model, a lag of the experimentally measured fronts is apparent relative to the front shape predicted by the constant speed model, depicted as a black line. **(B)** Data from the same experiments as shown in (A), but plotting the indent size Δ as function of front position *d* on a logarithmic scale (see Fig 2F). The slope of the data is consistent with the black line, which is the prediction of the constant speed model (Eq 1). **(C-D)** Comparison of the front shape (C) and indent size (D) between the numerical solution of the reaction-diffusion model (colored lines) and prediction of the constant speed model (black lines) for one obstacle. A lag, such as the one observed experimentally, is clearly visible, see also S5 Fig. **(E)** Snapshot of the numerical solution of the reaction-diffusion model. A traveling population wave encounters a region with zero growth (red rhombus). Population density is indicated in green, its profile far away from the obstacle is shown on the right. The white line indicates the inferred front. See S2 Video for the full solution and Materials and Methods for details.

The constant speed model also predicts that the shape of the front, scaled by the obstacle’s width, is identical for all rhombus-shaped obstacles. To test this prediction, we repeated the experiment for three more obstacles, in total combining two different widths with two different heights. Fig 3A and 3B display front shapes and the indent sizes as measured for all four obstacle shapes. As predicted, the data collapse very well onto single lines if lengths are divided by *w*. (This is not the case for other scalings, see S3 Fig.)

Although the constant speed model successfully predicts how the experimentally determined front shape (colored lines) scales with the obstacle’s width and the front’s distance from the obstacle, the experimental profiles display a lag relative to the predicted front (black line) for all four different obstacles and, equivalently, have a larger indent size than predicted (Fig 3A and 3B). However, this quantitative disagreement does not affect the scaling behavior of Δ(*d*) (Fig 3B, see S4 Fig for the same data on linear scale).

### Reaction-diffusion modeling rationalizes limitations of constant speed model

The constant speed model captures the general features of the front dynamics observed in the phage experiment, but the deviation prompted us to study a more detailed model which considers more of the details of phage propagation. In addition to understanding the deviation, the more detailed model will shed light on the range of applicability of the constant speed model.

The dynamics of plaque growth on *homogeneous* lawns has attracted considerable interest in the past [35–39]. A reaction-diffusion model, which captures the phage-bacterial interaction, the phage life cycle, and focuses on bacteriophage T7, has been suggested by Yin and McCaskill [36]: phage bind bacteria to form infected cells, and these, with a rate constant, burst to release more phage. More complex successor models focusing on the delay between infection and release of progeny phage have been published [38]. We decided not to generalize these models to heterogeneous environments for two reasons: (i) The appropriate parameters are not known for our experiments and (ii) we aimed for a general description that allows us to reach conclusions that extend beyond the infection of *E. coli* by bacteriophage T7.

We therefore employed a coarse-grained reaction-diffusion model in which a species disperses by diffusion and replicates locally with logistic growth (the local reproduction rate increases linearly with population density, then decreases and reaches zero at the carrying capacity of the environment) except inside of obstacles. In the absence of obstacles, this model produces a traveling population wave with an exponentially shaped leading edge that moves at constant speed like the population wave resulting from the model by Yin and McCaskill [36] (see Materials and Methods for a brief discussion of the differences and similarities between our model and phage population spread). Mathematically, it is a version of the Fisher-Kolmogoroff-Petrovsky-Piscounoff equation (FKPP equation) [40–43], which captures the two processes underlying a range expansion, dispersal and growth. In our generalized version, the growth function depends on location to include the effect of obstacles. The time evolution of phage population density *u*(**x**, *t*) depending on location **x** and time *t* is given by:

$$\frac{\partial u(x,t)}{\partial t}=D_{\text{eff}}\frac{\partial^{2}u\left(x,t\right)}{\partial x^{2}}+k_{\text{eff}}\left(x\right)u\left(x,t\right)\left(K-u(x,t)\right)\;,$$ (2)

where the first term describes dispersal by diffusion with an effective diffusion coefficient *D*_eff_. The second term captures local logistic growth with reproductive rate *k*_eff_(**x**) and constant carrying capacity *K*. By rescaling the phage density *u*(**x**, *t*), we can set *K* = 1 without loss of generality. In general, *k*_eff_ will depend on the bacterial density, the number of phage an infected bacterium releases (the burst size), the adsorption kinetics of the phage, the rate for lysis of infected host, etc. [36].

We used our data to estimate the values for the phage’s effective diffusion coefficient and effective reproductive rate on the lawn of susceptible bacteria, ${\hat{D}}_{\text{eff}}$ and ${\hat{k}}_{\text{eff}}$, respectively. For biologically relevant initial conditions, an unimpeded, linear population front moves forward with front speed $v=2\sqrt{{\hat{D}}_{\text{eff}}{\hat{k}}_{\text{eff}}}$ and front width parameter $\xi =\sqrt{{\hat{D}}_{\text{eff}}/{\hat{k}}_{\text{eff}}}$ [43]. The front propagation is governed by the dynamics at the leading edge, a behavior we expect for the phage system (see Materials and Methods for a more detailed comparison to the phage system).

From Fig 2A we find that the plaque front extends with a speed of about 0.2 mm/h. With a rough estimate of the diffusion coefficient of 0.0144 mm^2^/h (Refs. [36, 44], Materials and Methods) we can determine an effective growth rate of ${\hat{k}}_{\text{eff}}=0.7\;/h$ for the phage in our experiments. We assume that the phage’s diffusion coefficient inside the obstacle remains the same, but that no growth is possible due to the lack of susceptible bacteria, thus allowing us to set *k*_eff_(**x**) = 0 inside the obstacle and $k_{\text{eff}}\left(x\right)={\hat{k}}_{\text{eff}}$ otherwise. With the diffusion coefficient to be the same inside and outside the obstacle, individuals can diffuse into the obstacle, reminiscent of an absorbing boundary. We think this is the case in the experimental system as well, although it is possible that the effective diffusion coefficient differs slightly in the region with resistant bacteria from the region with susceptible bacteria.

We next numerically solved Eq 2 for the four different obstacles considered experimentally. Fig 3E displays two snapshots from the numerical solution of the wide obstacle (see S2 Video). To quantify front shape at the leading edge, we defined front position as the boundary at which the population density is larger than 5% of the carrying capacity (white line in Fig 3E, see Materials and Methods). Fig 3C displays the fronts. For the wide obstacle (and the three other obstacles, S5 Fig) we observe a lag of the front for the numerical solution (colored line) relative to the constant speed prediction (black line), in qualitative agreement with the experiments. This lag also manifests itself in an increased indent size (Fig 3D). To test sensitivity to the value of the diffusion coefficient, ${\hat{D}}_{\text{eff}}$, we repeated the analysis for the wide obstacle with ${\hat{D}}_{\text{eff}}\to 3{\hat{D}}_{\text{eff}}$ and ${\hat{D}}_{\text{eff}}\to {\hat{D}}_{\text{eff}}/3$ and found the lag to persist in both cases (S6 Fig, Materials and Methods). As expected, for decreasing ${\hat{D}}_{\text{eff}}$ the lag, relative to the constant speed prediction, becomes smaller. Taken together, the numerical solution of the reaction-diffusion model produces a lag similar to that seen in experiments of the phage model system (Fig 3) even though its parameters were not derived from the front’s shape.

Where does the lag originate from and under which circumstances is the constant speed model a good description? Both questions are closely related and can be explained by considering the relative importance of diffusion and movement of the front. While diffusion results in a mean distance traveled scaling with the square root of time, propagation of the front results in a position change of the edge of the front linear in time. In consequence, diffusion is the faster process at small length and time scales, while only propagation of the front leads to significant changes in population density at large length and time scales. The critical length dividing these two regimes is given by ${\hat{D}}_{\text{eff}}/v$, the ratio of the diffusion coefficient ${\hat{D}}_{\text{eff}}$ to the speed of the advancing front *v*. Up to a prefactor, this ratio is given by the front width parameter $\xi =\sqrt{{\hat{D}}_{\text{eff}}/{\hat{k}}_{\text{eff}}}$ and is proportional to the width of the profile, perpendicular to the front, which is shown in Fig 3E and S2 Video [43]. Small kinks in the front will eventually be rounded and small bulges in the front smoothed out by diffusion. (We disregard possible number fluctuations at the frontier and associated possible front instabilities [45].) The process of invasion, however, plays the major role in determining front shape on length scales much larger than *ξ*, justifying the use of the constant speed model as an approximate, but intuitively useful model for understanding how populations spread around obstacles. For our experimental system, *ξ* ≈ 0.1 mm which is considerably, but not strikingly, smaller than the scale determining the shape of the obstacle (1 − 2 mm).

The simplicity of the reaction-diffusion model (Eq 2 only has two free parameters.) allows us to identify two mechanisms for the lag of the front relative to the constant speed model: a modified shape of the front close to the obstacle’s boundary (S7 Fig, panel A) and a slow-down of the front around the point of maximum width (S7 Fig, panel B); see also S1 Appendix.

First, phage particles diffuse into the obstacle, recognizable by the obstacle in Fig 3E turning yellow at its boundaries. The obstacle is therefore partially absorbing and the phage sink leads to a reduced population density close to the boundary. This flux into the obstacle does not lead to a slow-down of the overall front, since the population extends far to the sides of the obstacle. Instead, a lagging boundary layer arises whose width is of the order of the only length scale, the front width parameter *ξ*, and which moves at the same speed as the unperturbed front (S7 Fig, panel A). If the obstacle induces large perturbations to the front (predicted by the constant speed model), this boundary layer will not be an important component of overall front shape. If the induced perturbation is small, however, the boundary layer becomes an important constituent of overall front shape. Because our obstacles are only one order of magnitude larger than *ξ*, we expect the lagging boundary layer to contribute to overall front shape and thus to the observed lag. (We attribute the differing shapes of the front at the boundary layer between experiment (Fig 3A) and theory (Fig 3C) to the coarse-graining embodied in our model and differences in front detection.) This effect will be modified if diffusion into the obstacle is not possible.

Second, expansions of circular populations with radii smaller than *ξ* are significantly slowed down compared to linear population fronts or circular population fronts with radii much larger than *ξ* [43]. The constant speed model predicts that a circular segment arises with a radius initially smaller than *ξ* when the front passes around the point of maximum width (Fig 2H, S7 Fig, panel B). A temporary slow-down is therefore expected until the radius becomes significantly larger than *ξ*, leading to an apparent lag of the front close to the obstacle. In general, we expect a contribution to lagging of the front wherever the boundary of the obstacle is kinked or curved (i.e., many infinitely small kinks are present).

Both effects depend on details of the obstacle’s shape, but are tied to the length scale *ξ*. The perturbations predicted by the model of constant speed, however, are tied to the size of the obstacle: doubling the size of the obstacle leads to a doubling of the size of the perturbation due to the obstacle. Both effects should therefore lead to only small corrections to the front shape predicted by the constant speed model in the limit of large obstacles (large in all directions, linear size *L* ≫ *ξ*).

### On large scales, the constant speed model predicts a universal front shape

Since we expect the constant speed model to successfully predict the front shape for large obstacles, we can construct the front shapes for more general obstacle shapes and infer general properties of front shape that are independent of the shape of the obstacle (see below and S1 Appendix), which is not possible using experiments or numerical solutions alone.

While for rhombus-shaped obstacles the front shape is particularly simple (the front consists of two linear and two circular segments only, Fig 2H), we now consider general convex, mirror-symmetric obstacles. When the front encounters an obstacle (as when it first envelops the tip of a rhombus or the front half of a circle), the shape of the front remains planar. As the obstacle starts to decrease in width, each point along the boundary is the source of a circular segment contributing to the front (similar to Fig 2E) and the front thus encounters the obstacle at a 90° angle. Eventually, a kink or a “cusp” (a kink with infinite slope) forms on the far side (S8 Fig), which heals downstream from the obstacle. Note that when changing the size of the obstacle (without changing its shape) the front’s overall shape stays unchanged, but gets scaled by the same factor that the obstacle size increased or decreased.

In addition, as the kink heals downstream from the obstacle, we eventually recover a scaling result similar to Eq 1. In this respect, the front exhibits a universal behavior far away from the obstacle: the perturbation inherited by the front is determined by the obstacle’s width, but not by its precise shape. Some quantitative predictions of the constant speed model for isolated circular, elliptical and elongated tilted obstacles are found in S1 Appendix (S8 and S9 Figs). For objects that are not convex, we expect a similar overall behavior. An obstacle with a complicated shape still results in a kink which gradually heals as the front moves beyond the obstacle (S10 Fig, S3 Video).

### Fate of genotypes is determined by their location relative to the obstacle

We next examine how genetic composition of a population is shaped by obstacles it encounters, first predicting the obstacle’s effects based on the constant speed model followed by examining an experimental model system and simulations. As populations expand, genetic drift leads to the local reduction of genetic diversity and the formation of monoclonal sections at the front [4]. Thus we consider a population front that contains different neutral genotypes at different positions along the front encountering an obstacle.

**Fig 4:**
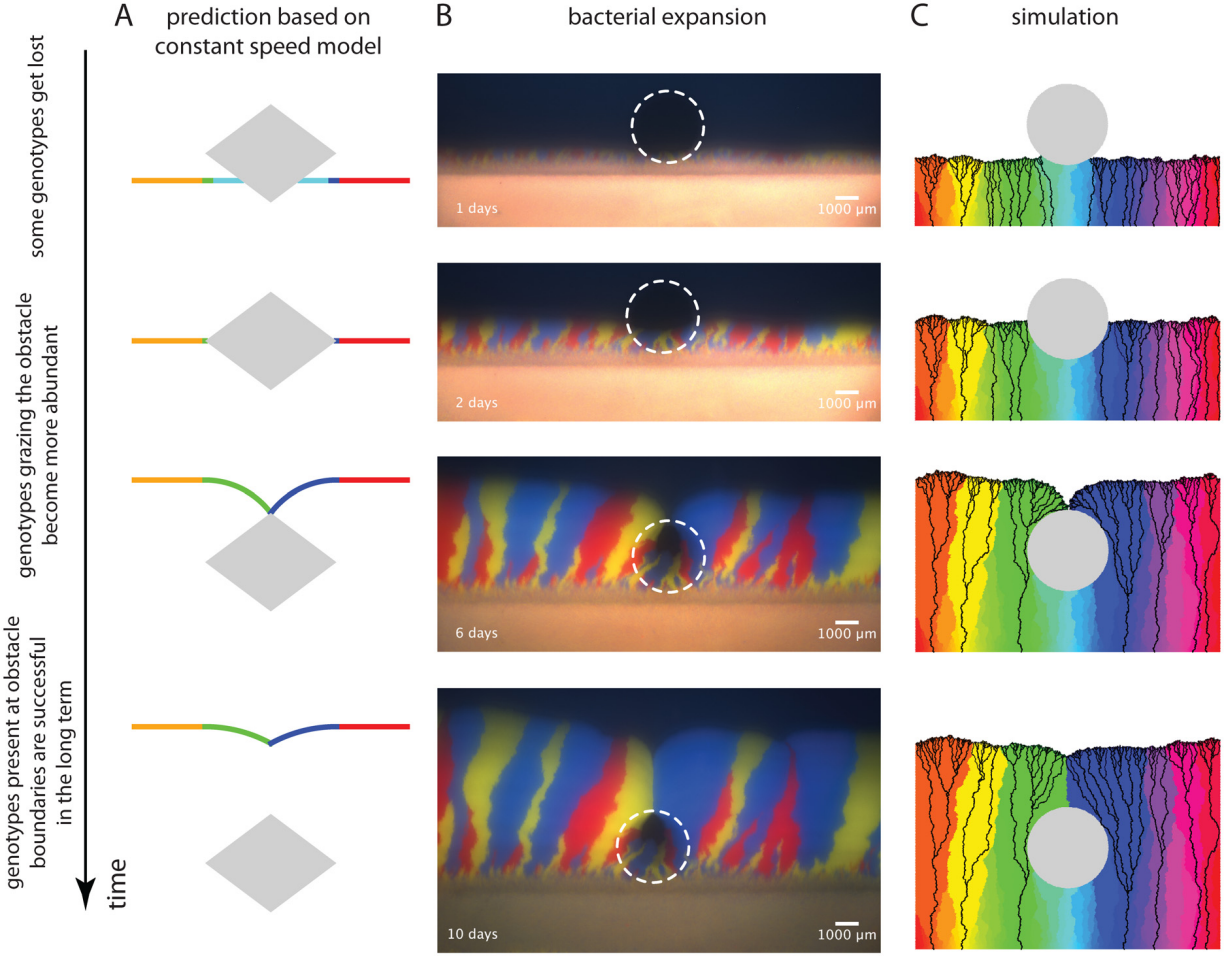
Effect of obstacle on genotype abundance at front. **(A)** Illustration of the changes in genotype abundance predicted by the constant speed model. Genotypes exactly at the frontier can be (i) driven to extinction (cyan), (ii) largely unaffected by the obstacle (orange & red), or (iii) sweep through a substantial part of the population after grazing the corners of the obstacle (green & blue). **(B)** Spatial expansion of *E. coli* with three different fluorescent labels on agar plates around a circular region with reduced nutrients (white dashed circle serves as guide to eye). In addition to the features outlined in the text, the sector boundary, where the two grazing sectors meet to form the kink in the population front, appears to be significantly more straight than the other sector boundaries in the colony. Finally, for the replicate shown here, the sector boundary behind the obstacle is aligned in the propagation direction of the front but the majority of the remaining sector boundaries display a slight bias to the right. The tilt to the right is equivalent to the chirality of sector boundaries seen for some *E. coli* strains in circular spatial expansions [13, 46] and the straight boundary behind the obstacle suggests that something is phenomenologically different when two sectors meet each other. See S4 Video for the complete time lapse information. **(C)** Results of a stochastic simulation of a lattice-based model with about 600 genotypes (colors) encountering a circular obstacle (gray area). Black lines are lineages together representing the genealogy of individuals at the final front position. See S5 Video for a depiction of the full simulation. The individual experimental replicate and simulation were chosen to highlight the features described in the main text; other replicates and simulations show the same overall behavior and are consistent with the description in the main text.

Fig 4A displays a series of front shapes together with a simplified initial genotype distribution indicated by orange, green, cyan, blue, and red colors. In the spirit of the constant speed model, we focus on front shape dynamics alone. The front segment with the cyan genotype either cannot propagate within the obstacle or, in the case of bacteriophage T7, slows down dramatically since only diffusive motion is possible. Hence, this genotype is lost and does not contribute to the range expansion at later times. After passing the point of maximum width, the circular arcs of the front in the ‘shadow’ of the obstacle grow due to inflation and therefore genotypes marked in green and blue occupy a larger part of the front. As the kink heals, the green and blue genotypes occupy the part of the frontier that lies in the shadow of the obstacle. The abundance of these genotypes, which were a small fraction of the initial front, stays elevated even after the kink has healed. Note, however, that part of the increase in genotype abundance is transient since the arc length of the circular segments gets reduced during healing of the kink, although the radius still grows and the front thus locally experiences inflation. Fig 4A depicts a special symmetric initial condition of genotype frequencies that guarantees that genotypes benefitting from the inflation in the wake of the obstacle (green and blue genotypes) will meet precisely at the top of the obstacle. However, selectively neutral, grazing genotypes will meet at the top for quite general initial conditions, i.e., there is always a boundary that gets ‘pinned’ at the top of the obstacle.

The constant speed model argues that genotypes that fail to encounter the obstacle will be unperturbed, those whose segment of the front entirely collides with the obstacle will be eliminated, and those that graze the obstacle will be privileged because they will fill in the region downstream of the obstacle.

We tested this idea experimentally by using fluorescent proteins as labels for selectively neutral genotypes. Because we could not produce expansions with fluorescent phage, we used the expansion of three *E. coli* strains, which express different fluorescent proteins. Two of the strains have been characterized previously [13] and we constructed a third strain which behaves comparably for the purpose of the experiment. We created heterogeneous agar plates by adding a circular membrane with an impermeable region just below the top layer of agar. We then launched linear expansions of mixtures of the three marked strains and observed them as they grew past the circle that blocked access to nutrients (Materials and Methods, S11 Fig). Fig 4B displays the range expansion after approximately 1, 2, 6, and 10 days of growth (see S4 Video for additional time points and Materials and Methods for a description of replicate experiments).

Before the population meets the obstacle, genetic drift at the population front leads to separation into monoclonal regions of the three different colors [13, 47]. After formation of these sectors, their boundaries wander which results in a coarsening of sectors [13]. Abstracting from this effect, we observe that the sectors encountering the obstacle head-on are lost but the two that just graze the obstacle grow in its shadow, increasing the abundance of the corresponding genotypes, and meet at the top of the obstacle. These features are experimentally reproducible and verify the predictions of the constant speed model (Materials and Methods).

Next, we performed stochastic simulations, in which individuals reproduce on a lattice. In each step, a site along the front is randomly chosen and is copied onto one of the unoccupied neighbored sites thus propagating the front [5] (a variant of the Eden model [48, 49] extended here to track genotypes, see S12 Fig and Materials and Methods). Individuals never die, i.e., occupied sites never change. The obstacle is a set of lattice sites which cannot be occupied. Fig 4C shows an initially linear front encountering an obstacle. The obstacle leads to dynamics that are qualitatively similar to the bacterial range expansion described above (S5 Video). Fig 4C illustrates that individual genotypes can go extinct by two processes: the wiggling of sector boundaries caused by genetic drift [5, 13, 47] and collision with the obstacle (light green to light blue sectors, Fig 4C). The genotypes that graze the corners that define the obstacle’s width dominate the curved part of the front during the subsequent inflationary phase (green and purple sectors, Fig 4C) and meet at the top of the obstacle.

The founder effect of individuals near the point of maximum width also dominates the population’s genealogy downstream of the obstacle. Black lines in Fig 4C represent lineages of individuals at the front. As already evident from the labeling of genotypes with colors, none of the lineages pass through the area in front of the obstacle. In addition, most of the individuals at the curved part of the front originate from a small number of ancestors near the point of maximum width. Strikingly, none of these lineages pass through the point where the two populations meet behind the obstacle. Despite the expansion of the green and purple sectors right before they encounter each other, the parts of the population which meet at the top of the obstacle have no descendants at the front at late times. This effect arises because the two sectors encounter each other (almost) head-on just behind the obstacle (Fig 4C). Although this effect does not manifest itself in the sectoring pattern we deduced from the constant speed model (Fig 4A), it can be understood within the framework of the model: In Fig 2H, blue lines indicate the position of a virtual marker at the front coinciding with the overall shape of lineages behind the population front. This suggests that the constant speed model may also be used to predict the evolutionary dynamics of a spreading population in more complex environments.

In summary, we found that the constant speed model used to describe the front shape of an expanding population can be used to understand the effects of an obstacle on the diversity of neutral genotypes in an expanding population. These include the loss of genotypes encountering the obstacle head-on and a founder effect from individuals present at the point of maximum width. Since the obstacle does not affect fitness of individuals carrying specific genotypes, but in an intricate way increases random fluctuations, these effects are an example of ‘geometry-enhanced genetic drift’.

### Expanding population wave encountering two nearby obstacles

**Fig 5:**
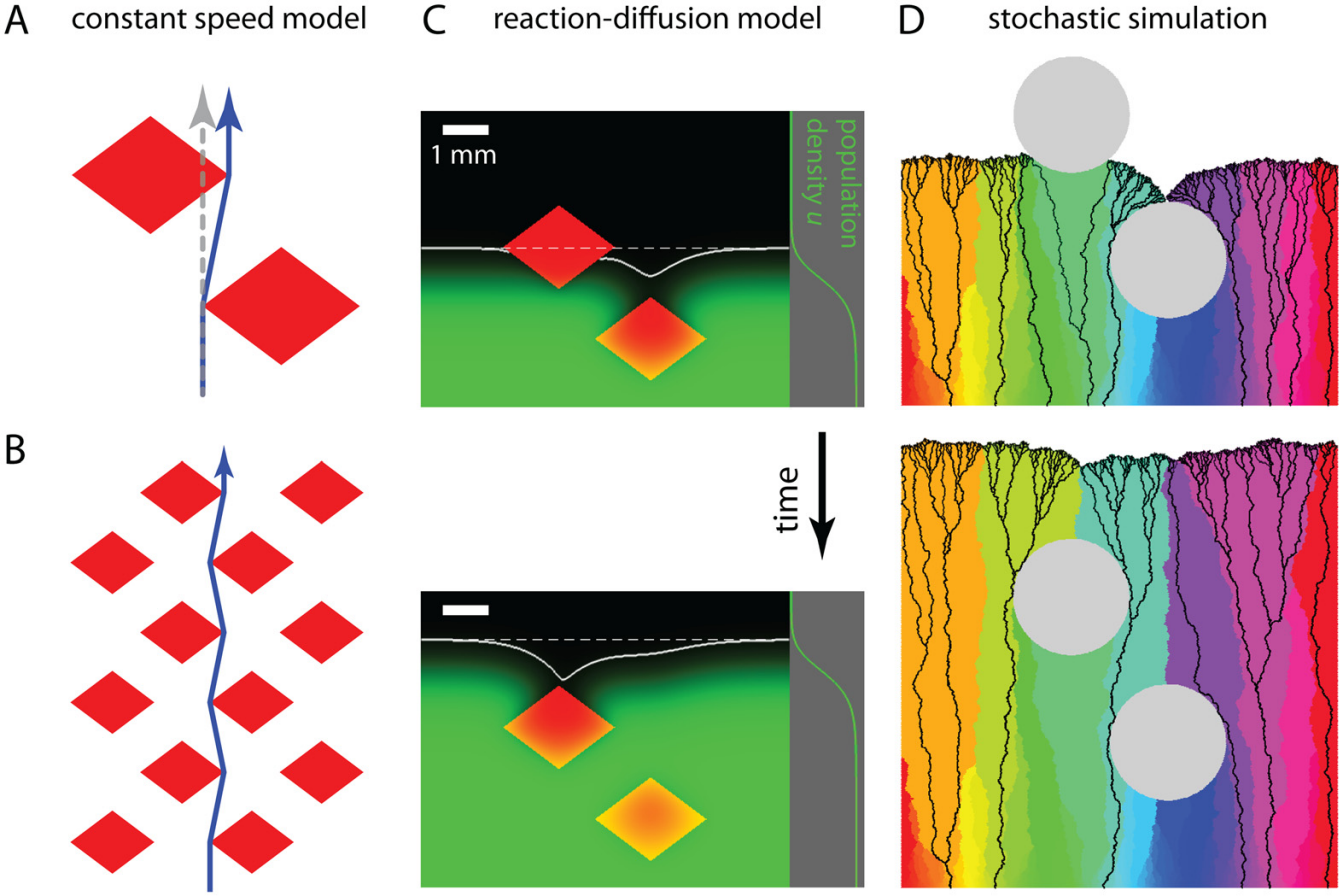
Effect of two obstacles slightly offset with respect to each other on front dynamics and genetic composition of the population. **(A)** Constant speed model prediction for a front passing between two obstacles (red shapes). The blue arrow, the path of a virtual marker (compare Fig 2H), indicates the shortest path passing in between the obstacles. The gray dashed arrow indicates the path of a virtual marker if the second obstacle was absent. **(B)** Extension to a regular lattice of rhombus-shaped obstacles, the blue arrow indicates the path of a virtual marker through the maze of obstacles. **(C)** Two snapshots of the numerical solution of the reaction-diffusion model in the presence of two obstacles (regions of zero growth, indicated in red). Population density is indicated in green (the profile far from the obstacles is depicted on the right). The white full line marks the inferred front and the dashed line depicts the position of the front far from the two obstacles. See S6 Video for the full solution and Materials and Methods for details. **(D)** Snapshots of a stochastic simulation of a population with initially about 600 genotypes encountering two obstacles, in analogy to the situation where the population is encountering one obstacle (see caption of Fig 4C and Materials & Methods for details and S7 Video for a depiction of the whole simulation).

In the regime in which the constant speed model is valid, the effect of the obstacle on front shape is limited to a downstream region as wide as the obstacle and is transient due to healing of the kink (Fig 2H). If the habitat is much larger than a single obstacle, the overall front speed and shape is therefore not influenced by the presence of a single obstacle. What is the effect of many such obstacles introduced in Fig 1A? Insight can be gained by considering two obstacles which are offset and placed behind each other as displayed in Fig 5A. We focus on the population front between both obstacles arguing that in the presence of many obstacles the population encounters such pairs of obstacles subsequently. Instead of displaying the front at different time points, a blue arrow is used to indicate the path of an imaginary marker at the front which propagates with constant speed (compare to Fig 2H; the path of the marker can be derived from minimizing path length as explained in S1 Appendix on the ‘Analogy to geometrical optics’). The dashed gray arrow indicates the path of that marker in the absence of the second obstacle illustrating that the presence of the second obstacle lengthens the path and thus slows down the front between both obstacles. This effect is more readily visible in a regular pattern of rhombus-shaped obstacles (Fig 5B); the path of the virtual marker repeatedly changes direction, the speed of the front in normal direction is lower. To address the same scenario using the reaction-diffusion model, we extended our analysis of Eq 2 using the parameters employed to study the case of a single obstacle (Fig 3E). Fig 5C displays two snapshots of the numerical solution (S6 Video, Materials and Methods). Both obstacles transiently perturb the front, but not independently. Due to the first obstacle, the front reaches the right side of the second obstacle after it reaches the left side, resulting in the formation of a kink which is asymmetric and slightly shifted to the right. The front lags the unperturbed part of the front (dashed white line indicating front position at the boundary of the channel) in the wake of both obstacles, effectively resulting in a slow-down. As discussed above, for the obstacle size considered, the constant speed model is not a perfect description of front shape. The lag observed relative to the unperturbed front therefore originates from a combination of the geometrical slow-down in Fig 5A and a slow-down for reasons discussed above.

Extending our qualitative analysis of ‘geometry-enhanced genetic drift’ we repeated the stochastic simulation (Fig 4C) with two obstacles. In Fig 5D two snapshots of one realization of the simulation are displayed. Due to the stochastic nature of genetic drift, rigorously analyzing the effects of multiple obstacles on genetic diversity is not possible without detailed quantification. However, we observe two effects expected from our understanding of single obstacles. First, there is a sector boundary at the top of the second obstacle with two sectors encountering each other from opposite sides of the obstacle. Second, there is a lineage passing the first obstacle on the left and the second obstacle on the right and just grazes both obstacles (see Materials and Methods for a discussion of other instances of the simulation). These two observations illustrate two of the effects we expect many obstacles to have on genetic diversity. First, a subset of sector boundaries will be created or pinned by obstacles introducing an effective wandering of sector boundaries not arising from genetic drift at the front (the mechanism for wandering of sector boundaries in the absence of obstacles). Second, if lineages preferentially graze sides of obstacles, an effective description of the genealogy in a complex environment may be possible by considering a small subset of possible paths through the maze of many obstacles.

## Discussion

Organisms rarely spread across featureless habitats. Instead, they must find ways to survive and reproduce in the presence of environments that are heterogeneous in space and time. To investigate the effects of spatial heterogeneities on the dynamics and genetics of a spreading population, we combined experimental and theoretical approaches to understand the effect of single obstacles, of defined geometry, where organisms could not reproduce. When bacteriophage T7 encounters resistant *E. coli* the bacteriophage population front is perturbed in the wake of the obstacles by a sharp kink that slowly heals as the front moves on. A constant speed model gives an intuitive understanding of this perturbation, and a more detailed reaction-diffusion model rationalizes the deviation between experiment and the constant speed model’s predictions. In addition, the constant speed model explains that in a genetically diverse population, genotypes that run into the obstacle are eliminated and those that graze its sides increase in abundance, an example of ‘geometry-enhanced genetic drift’.

A mathematically rigorous analysis by Berestycki et al. predicted transient perturbations of planar waves encountering a single compact obstacle [50]. From a physical perspective, when the obstacle’s linear size *L* satisfies *L* ≫ *ξ*, where *ξ* characterizes the front width, considerable understanding of the perturbation is possible using a model based on front propagation locally and with constant speed. In this limit, the shape of the front can be found using a straightforward geometric construction that has an analogy in geometrical optics (S1 Appendix). Interestingly, in this regime, a linear front stays unperturbed while it envelops the obstacle, in contrast to a first intuition based on a front of fluid material encountering an obstacle such as lava flow encountering a barrier [51]. However, a front of forest fire resembles the situation of phage propagating on a lawn of bacteria; indeed, ideas very similar to the model of constant speed are used to predict forest fires [52]. Our analysis of the front predicted by the constant speed model shows that the width of the obstacle, and not its precise shape, determines the long-term dynamics of the perturbation caused by the obstacle.

The study of two obstacles placed behind each other and offset suggests an overall slow-down of the front in the presence of many obstacles. This effect is expected to depend on the density of obstacles. If obstacles are sparse, the healing of the perturbations implies that the front speed should be only marginally reduced compared to expansion in the absence of any obstacles. If obstacles are close enough to each other that the perturbation from the preceding obstacle has not healed much before the next obstacle is encountered, the perturbations will add up faster and an ensemble of obstacles will reduce front speed more. Obstacles regularly placed on a lattice are a special case: the existence of open channels, unobstructed by obstacles and much wider than the front width parameter, will allow the front to travel as fast as it would without obstacles; the remaining territory will then be explored in the wake of the front.

If the density of obstacles is so high that no free paths connect the different boundaries of the environment, the traveling wave cannot propagate around obstacles. When dispersal within obstacles is possible, the population can nevertheless expand via migration between regions with good growth conditions, which is essentially the island scenario depicted in Fig 1A. Invasion is not possible in a scenario where population spread is hindered by a connected set of impermeable obstacles (compare to the percolation threshold concept [53]).

When the size of the obstacle approaches the parameter that sets the width of the population front, the constant speed model breaks down. This regime can be understood by numerically solving a two-dimensional reaction-diffusion system (a generalized FKPP equation), which rationalizes the lag between the experimentally observed phage front and the constant speed model prediction, and bridges the gap to the regime where the length scale of the heterogeneities in the environment is much smaller than *ξ* and perturbations in front shape are therefore not expected.

Following these ideas will complement recent studies using reaction-diffusion models to study invasion in heterogeneous environments [20]. From the experimental side, extending the printing assay to environments with many obstacles or creating random environments by spotting a mixture of bacteria susceptible and resistant to phage onto an agar surface (S8 Video, S13 Fig, Materials and Methods) might shed light on this question in the future.

The models we used to describe the spread of phage populations were successful, even though they ignored the details of the bacteriophage life cycle. We found that for large obstacles the constant speed model is a good description for the front shape and expect in consequence the effects of ‘geometry-enhanced genetic drift’ to hold. How do these results apply to organisms whose spreading mechanism is very complex or even not well characterized? In general we expect that for other population waves than those considered here, similar considerations hold. Specifically, we expect a length scale to exist beyond which a constant speed approximation results in a good description of front shape. Thus, our findings based on the constant speed model such as universality of the shape of the population front and the genetic consequences should be applicable to population fronts with a differing underlying dynamics, including pushed fronts (fronts where the bulk of the wave and not the leading edge sets the dynamics [24]). Upon decreasing the obstacle size, we expect the constant speed model to break down and front shape and population spread to depend on the details of the biological system considered.

Similar considerations hold when the nature of the heterogeneities is changed. We here considered obstacles with vanishing growth rate. If, however, the obstacle was a region with reflecting boundary conditions, i.e., diffusive dispersal into the obstacle was not possible, we expect the behavior on large scales for large obstacles to be described by the constant speed model, while the behavior at small scales and near the boundary of the obstacle would be different.

When the obstacle strongly perturbs the shape of the population front, we predicted that these perturbations affect the fate of genotypes and lead to ‘geometry-enhanced genetic drift’. Analyzing the fate of lineages shows that the descendants of individuals trapped in front of the obstacle or born right behind it are lost in the long term. Our results are in qualitative agreement with a simulation study that demonstrated a decreased probability of survival of neutral (and deleterious) mutations occurring just in front of and right behind an obstacle [27]. Taken together, our results show that the long-term reproductive success of an individual depends on its position relative to the obstacle the population encounters as well as the random sampling that drives genetic drift, expanding the list of factors that contribute to ‘survival of the luckiest’ [54]. In addition to these effects, obstacles separating two genotypes for a considerable amount of time could also help preserve genetic diversity, similar to the mechanism of allopatric speciation. Theory and simulation, including a more detailed description of the evolutionary dynamics on top of the population dynamics is needed to disentangle these effects.

More work is also needed to understand the effects of many obstacles: Considering the effects of two obstacles placed behind each other and offset illustrates that obstacles can shape the genetic composition of a population by creating transition zones between two genotypes and constraining the spatial structure of lineages. More research is needed to illuminate how genetic drift at the population front and ‘geometry-enhanced genetic drift’ due to obstacles together shape the genetic makeup of a population.

Single obstacles could have pronounced effects on evolution beyond shaping the abundance of neutral genotypes. Because the small subpopulation that grazes the obstacle expands spectacularly, obstacles could make it easier for deleterious mutations to survive. This expansion protects deleterious mutations from extinction [55] and could establish a subpopulation which is large enough to survive for a considerable amount of time. This time span might be long enough for a second, beneficial mutation to occur, which has implications for the crossing of fitness valleys, similar to the effects due to genetic drift at the front [56]. Because the obstacle is not a population bottleneck, failure to acquire such a second mutation does not lead to a reduced fitness of the population in the long-term: genotypes that passed further away from the obstacle would eventually spread sideways and extinguish the deleterious allele if it is not rescued by a second beneficial mutation. A true spatial bottleneck would have fixed the deleterious allele and thus reduced the (absolute) fitness of the whole population. The rapid evolution of phage should allow such questions to be addressed experimentally in the future.

Higher organisms differ in two important aspects from the *E. coli* system and the stochastic simulation, they generally are diploid or polyploid and their population is dynamic behind the front. While the consequences of obstacles on diploid organisms undergoing recombination are an important area for future research, our results are relevant for the evolutionary dynamics of mitochondrial DNA carried by diploids. Gene flow behind the front will blur the sector boundaries which are frozen in both our experiments and the stochastic simulation. However, this diffusive blurring is slow (scaling as the square root of time since it is a diffusive process) while the front advances more rapidly (linearly with time). Hence, the boundaries remain well-defined for some distance behind the wave [57].

This study focused on a regime where the front dynamics can be described by a model of constant speed. In this regime, the results are insensitive to details of the expansions and details of the obstacle shape. Assuming that the population front is subdivided into monoclonal regions, an effect of ‘geometry-enhanced genetic drift’ can be described which is closely connected to the dynamics of front shape. We believe that these findings carry over to a wide variety of population expansions and beyond the neutral evolutionary dynamics considered here. Finally, although our analysis focused on single obstacles, we believe that our findings can be extended to natural environments, which typically display more complex heterogeneities. As a first step, by using the findings for isolated obstacles we expect to be able to describe observables such as the effective front speed and an ‘effective genetic drift’ in environments with obstacles such as those displayed in Fig 1A.

## Materials and Methods

### Experimental procedures

#### Plasmids

To fluorescently label bacteria, we used three different plasmids which (only) differed in the ORF for the fluorescent protein and the immediately surrounding nucleotides. The vector is pTrc99A, which provides resistance against ampicillin and expresses the *lac* repressor [58]. The ORF of the fluorescent protein is cloned between the SacI and XbaI sites and is under control of the *trc* promoter, a hybrid of the *trpB* and *lac* promoters. This promoter provides IPTG inducibility of the expression of the fluorescent protein. However, throughout our experiments, we did not add IPTG to the medium since we found the background expression to be sufficient for our experiments. Two of the plasmids (encoding for fluorescent proteins CFP and venus YFP) were identical to those described by Hallatschek et al. [13], while the third (encoding for mCherry) was obtained by substituting the ORF.

#### Strains

*E. coli - heterogeneous bacterial lawn*. Two strains of *E. coli* were used to produce a heterogeneous lawn for bacteriophage T7. One strain, *E. coli* BW25113 (CGSC# 7636) is susceptible to T7 phage infection, the other strain is partially resistant by means of the deletion of the *waaC* gene (also known as the *rfaC* gene) [59] (JW3596-1, CGSC# 11805, part of the Keio collection [60]). (The product of the *waaC* gene is involved in the synthesis of the lipopolysaccharide whose recognition is essential for adsorption of the phage [59].) Both strains were obtained from the Coli Genetic Stock Center (CGSC, New Haven, CT). The susceptible strain was transformed with the plasmid expressing venus YFP (resulting in strain eWM43), the resistant strain was transformed with the plasmid expressing mCherry (resulting in strain eWM44).

*E. coli - bacterial expansion*. To study range expansions of bacteria with different genotypes, three strains of *E. coli* DH5*α* transformed with the plasmids described above were used. Two of the strains were identical to those used by Hallatschek et al. [13] (expressing CFP (named strain eWM282) and expressing venus YFP (named strain eWM284)) and had been shown to be selectively neutral. The third strain was obtained by transforming *E. coli* DH5*α* with the plasmid expressing mCherry (eWM40) and showed a comparable front speed when grown together with the two other strains (see Fig 4B).

**Bacteriophage T7.** We originally obtained bacteriophage T7 as an aliquot from the wild-type stock of the Richardson laboratory (Harvard Medical School, Boston, MA). To obtain phage optimized for plaque spreading, phage was picked from the rim of a plaque growing on a top agar lawn of *E. coli* of the BW25113 background and subsequently grown in liquid *E. coli* culture of the BW25113 background. The lysate was mixed with NaCl to a final concentration of 1.4 M. The supernatant of the subsequent centrifugation step was stored at 4° C. Aliquots of this stock were used for all experiments.

#### Plaque growth around obstacles of defined size and shape

A detailed protocol for printing the bacterial lawn is presented in S1 Protocol. In short, agar patches were prepared by placing a 3.5 × 2 cm^2^ piece of nitrocellulose membrane (Millipore, 0.8 μm AAWP) onto solid agar, covering it with warm molten agar (2xYT medium with 20 g/l agar and 100 μg/ml ampicillin) and keeping it at room temperature for two nights. Plates were then refrigerated if they were to be used later. The top layer of agar was cut out together with the supporting membrane and placed onto the CD tray of an inkjet printer (Epson Artisan 50) immediately before the printing process (S1 Fig).

Overnight cultures of *E. coli* were grown at 37° C from single colonies in 2xYT with 100 μg/ml ampicillin. 5 ml (strain eWM43) and 15 ml (strain eWM44) of overnight culture were spun down and resuspended in 15 ml 42% glycerol, which was used to fill pristine refillable ink cartridges. (Similarly, cartridges were filled (or refilled) with 70% ethanol or deionized water and used for cleaning or flushing the printhead in the course of the printing process.) The TIF viewer IrfanView in conjunction with the Epson printer driver was used to print the pattern (CMYK TIF image) onto agar patches, twice for each of the four obstacle shapes. The resulting eight agar patches were then transferred to square plates (same medium as used for agar patches; see S1 Fig) and incubated at 37° C for roughly 5 hours. Additional steps allowed us to gauge the sterility and quality of the printing process (see S1 Protocol).

At this point, the outline of the obstacle was visible by eye and a linear stretch of phage was inoculated close to the obstacle region using a strip of nitrocellulose membrane (Millipore, 0.8 μm AAWP) soaked with solution of phage stock. (We ensured that no drops were visible on the membrane by stripping the membrane on a sterile surface and/or waiting till the liquid evaporated.) The plate was sealed using Parafilm and the individual regions were imaged using a stereomicroscope (Zeiss SteREO Lumar.V12) inside an custom-built incubator box at 37° C every 20 minutes for at least 24 hours. During the incubation, the Parafilm broke regularly, but the agar surface was not visibly impaired by cracks. Experiments were repeated as biological replicates on three different days with two technical replicates per obstacle shape as noted above (with one exception where placement of the phage-soaked membrane failed). Fig 2A and 2B show the front position and front shape for the replicates of one of the four different obstacle shapes, Fig 3A and 3B display the front shape and kink size for all replicates considered. We regularly observed lysis of bacteria missing the gene *waaC* (red fluorescence marker). This phenomenon occured in the wake of the primary front, which is of interest here, and therefore does not affect the interpretation and analysis of our experiments.

#### Spatial expansion of *E. coli* around regions with poor nutrient conditions

Plates with a region of lower nutrient concentrations for *E. coli* were prepared as sketched in S11 Fig: Nitrocellulose membranes (Millipore, 0.8 μm AAWP) with small impermeable regions were created using the Sylgard 184 Silicone Elastomer Kit. A drop of base mixed with curing agent was placed onto the membrane and subsequently cured. (Membranes can be sterilized afterwards using UV light.) Membranes were placed between two layers of medium (2xYT with agar, see below). To that end, 25 ml medium were pipetted into empty standard plates (diameter 8.5 cm). The prepared membrane was placed onto the solidified agar and covered with 2 ml of molten agar and distributed as uniformly as possible. Plates were used after two nights at room temperature.

Bacteria (strains eWM282, eWM284, eWM40) were grown overnight from a lawn or from single colonies in 2xYT with 100 μg/ml ampicillin. Next day, cultures from the previous day were directly mixed or mixed after tenfold dilution in water at a ratio of 1:1:1. A linear inoculation was achieved by soaking a small piece of membrane with a linear edge in the bacterial solution which was then placed onto the agar surface. The plates were incubated at 37° C in a closed container which also contained a beaker filled with water to increase humidity. Plates were imaged regularly using a stereomicroscope (Zeiss SteREO Lumar.V12) in the three fluorescent channels corresponding to the fluorescent proteins used to mark the cells. For analysis, we only considered those experiments where the *E. coli* colony did not completely cover the region with poorer growth condition. The experiments satisfying this criterion were from three different biological replicates, had an unfavorable region with a diameter of between 2.0 and 3.5 mm and the agar concentration was 10, 12.5 or 20 g/l and ampicillin was absent or at a concentration of 100 μg/ml. All displayed the boost in genotype abundance behind the obstacle, sectors meeting just behind the obstacle, and a straight sector boundary behind the obstacle. When individual sectors were visible at the point the front encountered the obstacle, sectors encountering the obstacle head-on were lost.

#### Plaque growth in randomly structured heterogeneous environments

Plates (outer measure: 86 mm × 128 mm) were filled with 40 ml media (2xYT, 20 g/l agar and 100 μg/ml ampicillin) and kept at room temperature for two nights after pouring. Plates that were not used immediately after this drying period were then refrigerated and used at a later point. Bacteria strains eWM43 and eWM44 were grown overnight from single colonies in 2xYT with ampicillin (100 μg/ml), diluted 10^5^-fold in 2xYT with ampicillin and mixed in a ratio of eWM44:eWM43 = 3:1. For the control experiments without the resistant strain, the dilution of eWM44 was substituted by 2xYT with ampicillin. Four technical replicates of the mixture and two technical replicates of the control (5 μl each) were spotted onto the plate and incubated at 37° C. After roughly 18 hours, phage T7 was added using a pipette tip which was dipped into an aliquot of phage stock. The plate was sealed with Parafilm and individual colonies were continuously imaged using transmitted brightfield and fluorescence every 20 min for 26 hours using a stereomicroscope (Zeiss SteREO Lumar.V12). To provide a constant temperature environment, imaging was carried out in a custom-built enclosure kept at 37° C. During the imaging time course, the Parafilm broke, impairing the sealing, but plates did not dry out significantly. This experiment was done in three biological replicates (and technical replicates thereof). All replicates showed the behavior described in the caption of S8 Video. In addition, as is visible in S8 Video for example, lysis of bacteria with red fluorescent marker is regularly visible in the wake of the primary phage front. This is probably due to phage mutants which can infect *E. coli* missing the gene *waaC*.

### Image analysis of bacteriophage T7 expansions

A semi-automated image analysis pipeline was used to extract front shapes, front positions, and indent sizes (such as in Figs 2A, 2B, 3A and 3B) from the fluorescence time-lapse information. First, the channel detecting YFP fluorescence was used to define a front right after the plaque boundary got established and the channel detecting mCherry was used to identify the three upper corners of the obstacle. This information was used to define a coordinate system with the obstacle’s center at the origin and the front extending in *y*-direction (referred to as ‘upper region’ in the following, e.g., Fig 1D). The image was cropped (5.3 mm in direction of front movement, 0.7 mm in direction opposite to front movement, and 3 mm to either side of the obstacle). After normalization using the upper, uninfected region, the difference between two consecutive frames (YFP channel) was used to identify the front. In the difference image the extending front manifests itself as a bright region whose upper boundary was identified using thresholding. The algorithm was tested manually since the front is easily detectable by eye, although the decay in fluorescence extends to about 1 mm (S2 Fig). A few frames were excluded from the analysis due to jumps in the front which could be detected automatically using a threshold for local slope of front shape. Finally, front position was determined from the curve of the front close to the boundaries of the cropped region. Indent size was derived as the distance between the most lagging part of the front and front position (after the kink has formed, i.e., curve of front was defined around the axis of bilateral symmetry). The corners of the obstacles identified were also used to identify the size of obstacles, which were slightly smaller than in the printing template. When displaying data for individual obstacles the median of all the obstacles included in the analysis was used; for collapse plots, the size of each single obstacle was used to rescale data. For analysis, front detection was limited to frames obtained within 22 h even if the experiment lasted longer. After this time front detection becomes more challenging most likely due to the bacterial lawn transitioning into stationary phase.

### Numerical methods and simulations

#### Generalized Fisher-Kolmogoroff equation—model and parameter choices

To test the generality of the experimentally observed front perturbations and to investigate the lag between the constant speed model prediction and the observed plaque boundary, a reaction-diffusion system was employed, specifically a generalized Fisher-Kolmogoroff-Petrovsky-Piscounoff equation (FKPP equation) [40–43] as outlined in the main text. The FKPP equation was chosen despite the existence of more complex microscopic models for phage spread [35–39] since it is readily parametrizable. As outlined in the main text, two parameters of the FKPP equation,

$$\frac{\partial u(x,t)}{\partial t}=D_{\text{eff}}\frac{\partial^{2}u\left(x,t\right)}{\partial x^{2}}+k_{\text{eff}}\left(x\right)u\left(x,t\right)\left(K-u(x,t)\right)\;,$$ (3)

the effective diffusion coefficient $D_{\text{eff}}={\hat{D}}_{\text{eff}}$ and the effective growth rate $k_{\text{eff}}\left(x\right)={\hat{k}}_{\text{eff}}$ specify the front speed via $v=2\sqrt{{\hat{D}}_{\text{eff}}{\hat{k}}_{\text{eff}}}$ [43] in the case of a homogeneous environment (in our case a homogeneous lawn of susceptible bacteria) and a planar population front. (By rescaling the concentration field *u*(**x**, *t*), we can set *K* = 1 without loss of generality.) Conversely, with the front speed determined experimentally, it suffices to know either ${\hat{D}}_{\text{eff}}$ or ${\hat{k}}_{\text{eff}}$ to fully parameterize the model for a homogeneous environment and a planar front. We therefore estimated ${\hat{D}}_{\text{eff}}$ and used front speed far away from the obstacle (*v* ≈ 0.2 mm/h, Fig 2A) to specify $k_{\text{eff}}\left(x\right)={\hat{k}}_{\text{eff}}$ outside the obstacle and set *k*_eff_(**x**) = 0 inside the obstacle.

The diffusion coefficient of the phage has not been measured directly for the experimental conditions we employed. Depending on whether the diffusion predominantly occurs in the agar, in the bacterial lawn or in a layer of liquid, the diffusion coefficient is determined by the mesh size of the agar, the density of bacterial cells in the lawn and the humidity. We estimated the diffusion coefficient as follows: First, the diffusion coefficient of phage P22 has been used as a proxy for T7 diffusion [36]; in 10 g/l agar *D*_P22_ ≈ 4 ⋅ 10^−8^ cm^2^/s = 0.0144 mm^2^/h [44]. Second, as an upper bound, we can estimate the diffusion coefficient in water with the phage represented by a sphere with a diameter of 60 nm and a viscosity of 0.692 mPas for water at 37° C (source: wolframalpha.com) which results in a diffusion coefficient of *D*_SE_ = 0.04 mm^2^/h, about a factor three larger than *D*_P22_. Most likely, however, the true diffusion coefficient is smaller than *D*_P22_ since the agar concentration in our experiments is higher (20 g/l) and bacteria will restrict diffusion to interstitial regions (see discussion in Ref. [36]). We therefore also considered a diffusion coefficient three times smaller than for bacteriophage P22 in agar as a third case. In all three cases, the front lags behind the prediction of the model of constant speed, see S6 Fig. As expected, for a smaller diffusion coefficient, the lag becomes smaller since the obstacle becomes larger relative to the front width ($∼\sqrt{{\hat{D}}_{\text{eff}}/{\hat{k}}_{\text{eff}}}$) and the constant speed model becomes more accurate. An even smaller value of ${\hat{D}}_{\text{eff}}$ would therefore suggest even better agreement with the prediction of the model of constant speed. Since for our experiments, the value for ${\hat{D}}_{\text{eff}}$ is not known, we chose ${\hat{D}}_{\text{eff}}\approx 4\cdot 10^{-8}\;{\text{cm}}^{2}/s=0.0144\;{\text{mm}}^{2}/h$ and therefore ${\hat{k}}_{\text{eff}}=0.7\;/h$ throughout this work. Note that, although the bacterial lawn is changing throughout the experiment (bacteria are presumably not in stationary phase yet), the front speed is roughly constant (Fig 2A) indicating that small changes in parameters only weakly affect the front dynamics, justifying our coarse-grained approach with these parameter estimates.

Finally, let us note qualitative differences and similarities between the reaction-diffusion model used here and the phage system: (i) The heterogeneous bacterial lawn enters as a location-dependent parameter of an effective growth rate rather than a heterogeneous initial condition of bacterial density. (ii) While in the coarse-grained model a reduction in density below the carrying capacity due to outward-migration induces growth (Eq 3), this is not the case for the phage system where growth is not possible in the wake of the front. (iii) The FKPP equation with the logistic growth term used here leads to a front governed by the dynamics at the leading edge, the same behavior as we expect for the phage system. (Waves with this behavior are called pulled waves [24].)

#### Generalized Fisher-Kolmogoroff equation—numerical solution

The generalized FKPP equation with a position-dependent growth rate representing obstacles was solved on a square lattice, with periodic boundary conditions along the directions perpendicular to the front on either side of the obstacle and fixed boundary conditions of 1 and 0 well behind and well in front of the obstacle, respectively. The lattice spacing was chosen to be $h=0.15\sqrt{{\hat{D}}_{\text{eff}}/{\hat{k}}_{\text{eff}}}$. The diffusion operator was discretized using a nine-point stencil (with lattice along *x* and *y* directions), Δ*u*(*x*, *y*) ≈ [−20*u*(*x*, *y*) + (*u*(*x* + *h*, *y* + *h*) + *u*(*x* + *h*, *y* − *h*) + *u*(*x* − *h*, *y* + *h*) + *u*(*x* − *h*, *y* − *h*)) + 4(*u*(*x*, *y* + *h*) + *u*(*x*, *y* − *h*) + *u*(*x* + *h*, *y*) + *u*(*x* − *h*, *y*))]/(6*h* ^2^), resulting in a system of ordinary differential equations.

As initial condition, a linear front profile was chosen, which subsequently developed into the profile for a Fisher wave of width $∼\sqrt{{\hat{D}}_{\text{eff}}/{\hat{k}}_{\text{eff}}}$ before encountering the obstacle (S2 Video, S14 Fig). The system of ordinary differential equations was solved using the solver ode113 in MATLAB. The front was defined by a population density threshold of *u*_thresh_ = 0.05 (outside the obstacle) with the goal of capturing the edge of the front in a robust manner. (In parallel, we determined the front using *u*_thresh_ = 0.5. In all cases considered, the front determined using this criterion also showed a lag with respect to the constant speed model, but subsequent analysis was limited to *u*_thresh_ = 0.05.) Front position was defined as the position of the front at the boundaries of the lattice, i.e., far away from the obstacle on either side in the *x* coordinate. To study the effect of a rather irregular obstacle on the shape of the front, the FKPP equation was solved for a mirror-symmetric, non-convex obstacle (S10 Fig and S3 Video). To mimic the obstacles considered in the experiments, the FKPP equation was solved for rhombus-shaped obstacles with (*w* = 0.9 mm, *h* = 0.9 mm), (*w* = 0.9 mm, *h* = 1.2 mm), (*w* = 1.2 mm, *h* = 0.9 mm), and (*w* = 1.2 mm, *h* = 1.2 mm) as well as for two obstacles with (*w* = 1.2 mm, *h* = 0.9 mm) offset by 2 mm in horizontal and 2 mm in vertical direction, respectively. For the obstacle with (*w* = 1.2 mm, *h* = 1.2 mm) the lattice spacing, the accuracy requirement of the algorithm, the distance of the boundaries from the obstacle center, and the time until the population wave reaches the obstacle had no influence on front shape.

#### Stochastic simulation of expansion on a hexagonal lattice of sites

To study the effect of the obstacle on genotype abundance in the presence of genetic drift, we employed a stochastic model of population growth on a hexagonal lattice, specifically a set of sites with zero or one individuals per site and stochastic growth onto neighboring sites (see, e.g., [5, 49]). A lattice model where the shape of the front has no undulations simplifies analytical calculations [5], but is not applicable here since the shape of the front is a priori unknown. We therefore generalized the model of unconstrained growth in two dimensions as described by Korolev et al. (Fig. 2a in Ref. [5]) to more than two genotypes, see S12 Fig for a detailed description. The obstacle was realized by withdrawing the ability to serve as empty or inhabitable lattice sites for a set of lattice sites (marked in gray in Figs 4C and S12). The same holds for the boundaries perpendicular to the overall front movement. (We did not choose periodic boundary conditions to simplify the illustration of the genealogy.) The system width was chosen to be approximately 600 times and the obstacle’s radius to be about approximately 80 times the distance between adjacent lattice sites, respectively. The obstacle center was placed one third of the system width ahead from the originally populated lattice sites and in the middle between the two boundaries to the left and right. One occupied row on the hexagonal lattice with all unique genotypes (i.e., colors) served as initial condition. In the course of the simulation the front with these rules roughens somewhat, an aspect which is not of central interest here. The genealogy of all occupied lattice sites with at least one free neighboring lattice site was determined by keeping track of the history of the simulation at different points in time.

We set up ten instances of the simulation and interpreted the results qualitatively. The following features were clearly visible in frames which included the genealogy for all instances or for the majority of instances: dynamics of the front as described throughout this paper, the loss of genotypes encountering the obstacle head-on, an increased abundance in the shade of the obstacle of genotypes grazing the obstacles, two sectors meeting at top of the obstacle, the lineages from individuals at the curved front originating from around the point of maximum width, and the lineages not passing through the point at the top of the obstacle at larger times.

We repeated the simulation for two obstacles with the same initial condition. Both obstacles are horizontally offset by 5/3 and vertically offset by 5/2 of the obstacle radius, respectively. We again interpreted the results based on ten instances of the simulation: In all ten instances we find a sector boundary at top of the second obstacle where sectors from both sides of the obstacle encounter each other. Furthermore, at the end of the simulation (lower panel of Fig 5D), there is a lineage which passes the first obstacle on the left and the second obstacle on the right in all cases. The lineage grazes both obstacles in about half of the ten instances the simulation was run.

## Supporting Information

S1 Video Time-lapse movie of plaque extension (dark region) on a printed lawn of susceptible bacteria (yellow) in the presence of an obstacle, a region of resistant bacteria (red). (For snapshots see Fig 1D.)
(AVI)

S2 Video Visualization of the numerical solution of the reaction-diffusion model for an rhombus-shaped obstacle with *w* = 1.2 mm and *h* = 0.9 mm (scale bar: 1 mm). The obstacle, region of growth rate *k*_eff_(**x**) = 0, is indicated in red, population density *u*(**x**) is indicated in green. On the right, the profile of the front on the left and right boundary of the lattice is shown. Scale bar represents 1 mm. (For snapshots see Fig 3E.)
(AVI)

S3 Video Visualization of the numerical solution of the reaction-diffusion model for an obstacle with a more complex shape. For details, see caption of S2 Video, for snapshots see S10 Fig.
(AVI)

S4 Video Expansion of an *E. coli* colony with three different strains with three different fluorescent markers around a region with poorer growth conditions over the course of 13 days. (For snapshots see Fig 4B.)
(AVI)

S5 Video Visualization of a stochastic simulation of a population with neutral genotypes in the presence of an obstacle. The obstacle is denoted by a gray circle, initially about 600 genotypes are present. Black lines in the last frame (and the snapshots in Fig 4C) represent lineages of lattice sites at the front.
(AVI)

S6 Video Visualization of the numerical solution of the reaction-diffusion model for two rhombus-shaped obstacles. For details, see caption of S2 Video, the additional dashed white line marks the position of the front at the boundary of the lattice. For snapshots see Fig 5C.
(AVI)

S7 Video Visualization of a stochastic simulation of a population with neutral genotypes in the presence of two obstacles. For details see caption of S5 Video, for snapshots see Fig 5D.
(AVI)

S8 Video Time-lapse movie of phage spread in heterogeneous environment. Yellow regions represent a bacterial lawn of *E. coli* cells susceptible to infection with bacteriophage T7 while red regions represent resistant *E. coli* cells. The plaque (dark region due to lysis of bacteria) is expanding on the lawn of *E. coli*. Randomly distributed heterogeneity was produced by inoculating a droplet of a dilute mixture of susceptible and resistant *E. coli* onto an agar plate. Each cell grew into a microcolony, resulting in a bacterial lawn that consists of patches of susceptible or resistant bacteria. After inoculating a drop of phage T7 at a discrete location, the phage population spread on this heterogeneous lawn. Approximately 12 hours after inoculating the phage, the plaque has extended halfway through the bacterial lawn. The phage front leaves behind a region of debris, through which the patches of resistant bacteria expand further. The front shows several undulations due to patches of resistant bacteria, absent in the control experiment lacking the resistant cells (S9 Video). The overall speed of the plaque front is determined by additional parameters, which likely include the density of bacterial cells and their metabolic state: the front moves faster at the rim of the lawn both in the presence and absence of obstacles (S8 Video and S9 Video). See S13 Fig for snapshots of fluorescence channels only. More details are found in Materials and Methods.
(AVI)

S9 Video Control experiment for phage spread in heterogeneous environment. Like S8 Video, but without patches of resistant cells. The shape of the plaque is much more regular.
(AVI)

S1 Appendix The constant speed model: Additional results, analogy to geometrical optics, and limitations. (PDF)

S1 Protocol Detailed protocol including general considerations for printing the bacterial lawn. (PDF)

S1 Fig Illustration of the printing protocol. **(A)** First, agar patches are created by placing a membrane (cut to 3.5 × 2 mm^2^) between two layers of agar. The membrane with the top layer of agar is cut out and placed onto the printer’s CD tray. A split CD is used to define position and ensure proper functioning of the printer. After printing of the bacterial solution, the agar patches are transferred to a larger agar plate which then is incubated. **(B)** Photograph of the CD tray with two agar patches loaded into the printer (Epson Artisan 50). **(C)** Image of the pattern used to print bacteria onto the agar patch. Susceptible bacteria are found in the yellow cartridge, resistant bacteria in the black cartridge which after printing leads to the pattern described in Fig 1C. **(D)** Picture of the plate with experiments on plaque growth around single obstacles, after incubation and imaging.
(TIF)

S2 Fig Profile of fluorescence intensity in the channel representing the cells susceptible to bacteriophage T7 infection, 3 mm to left and right of the center of the rhombus-shaped obstacle in the co-moving frame (where the front position is to be at 0) for the experiment displayed in Fig 1D and S1 Video. Different colors represent different time points where yellow corresponds to late times after the start of the experiment. Different front profiles do not collapse onto each other perfectly (and do so less well in other experiments), but the small differences are not expected to influence our results on front shape since algorithmic front detection was checked manually.
(TIF)

S3 Fig Experimentally determined front shapes from replicates for different obstacle shapes as indicated by color. Axes are not scaled or scaled by half the obstacle width, *w*, or half the obstacle height, *h*, as indicated (see also Fig 3A). Collapse is best when all lengths are measured in units of *w* (panel D, Fig 3A) as predicted by model of constant speed. The collapse of front shapes for obstacles of same width (but not same height) in panel (A) illustrates that width, not height, of the obstacle determines front shape for rhombus-shaped obstacles.
(TIF)

S4 Fig Indent size Δ as a function of front position *d* for both experiments and the numerical solution of the reaction-diffusion model for different types of obstacles specified below by color. The experimental data are scaled by half the obstacle width, *w*, compare Fig 3B and 3D of the main text.
(TIF)

S5 Fig Front shape in the reaction-diffusion model for different obstacle shapes (colored line) and the model of constant speed (black line), see Fig 3C. In all cases, the front in the reaction-diffusion model lags behind the front in the model of constant speed.
(TIF)

S6 FigFront shape in reaction-diffusion model with different effective diffusion coefficients (colored lines) and model of constant speed (black line).(Choice of diffusion coefficient determines effective growth rate as explained in the main text.) Green: ${\hat{D}}_{\text{eff}}=0.0144\;{\text{mm}}^{2}/h$, red: ${\hat{D}}_{\text{eff}}\to {\hat{D}}_{\text{eff}}/3$, blue: ${\hat{D}}_{\text{eff}}\to 3{\hat{D}}_{\text{eff}}$, see Materials and Methods for justification of the choices for ${\hat{D}}_{\text{eff}}$. In all cases, the front in the reaction-diffusion model lags behind the front predicted by the model of constant speed. The reduction of the lag with decreasing ${\hat{D}}_{\text{eff}}$ is consistent with a decrease in $\xi =\sqrt{{\hat{D}}_{\text{eff}}/{\hat{k}}_{\text{eff}}}$ which characterizes the limit of the model of constant front speed.(TIF)

S7 FigMechanisms for a lag of the front.
**(A)** Diffusion of the reproducing phage into the obstacle leads to a lagging front due to a boundary layer of width $∼\xi =\sqrt{{\hat{D}}_{\text{eff}}/{\hat{k}}_{\text{eff}}}$, where ${\hat{D}}_{\text{eff}}$ is the diffusion coefficient inside and outside the obstacle and ${\hat{k}}_{\text{eff}}$ the growth rate outside the obstacle. **(B)** A (rapid) change in the slope of an obstacle boundary can induce a lag while the radius *r* of the circular segments of the population front is smaller than the characteristic length scale *ξ*, r≲ξ. See text and S1 Appendix for details.(TIF)

S8 Fig Geometric construction of the front behind a circular obstacle of radius *r* using the constant speed model, (from left to right) before the kink forms, when the kink forms from a cusp with infinite vertical slope and as the kink heals. At any given time, the front in the shadow of the obstacle is composed of infinitely many circular segments with centers at the boundary of the circle. Since the upper boundary of the obstacle is parallel to the original front, the opening angle *ϕ* of the kink vanishes at the moment the kink forms, resulting in two locally parallel population fronts. (The kink therefore is a cusp in this case.) As the kink heals, circular inflation still occurs locally, but the arc length of the perturbed front is reduced. At large times during the healing phase fewer and fewer circular segments contribute to the front: Finally, only those with centers close to the point of maximum width are relevant. In this long time, large distance limit, the detailed shape of the obstacle drops out. In addition, the coordinate system and the parameter *θ* used to describe the circle are indicated.
(TIF)

S9 Fig Illustration of the front shape for a tilted, very thin obstacle, which is indicated by the red line. The projected width of the obstacle is 2*L*, the obstacle is tilted by an angle arctan(*h*/*L*). Black segments of circles centered on the edges of the obstacle indicate front shape at a given point during the healing process. The kink forms off-center, but its tip approaches a line normal to the unperturbed population front that bisects the projected obstacle (curved blue line). See S1 Appendix for details.
(TIF)

S10 Fig Snapshots of the numerical solution of the reaction-diffusion model with a region of no growth with complex shape. The region of no growth is indicated in red. Population density is indicated in green, the inferred front of the traveling population wave is marked white. See S3 Video for all frames and compare to Fig 3E for a rhombus-shaped obstacle.
(TIF)

S11 Fig Experimental setup for bacterial expansions in the presence of a region with poorer growth conditions. **(A)** A part of a membrane is first made impermeable (see Materials & Methods). The membrane is then placed on top of an agar layer and covered by a thin layer of agar. **(B)** Picture of plate after bacterial expansion took place.
(TIF)

S12 Fig Illustration of the algorithm for the stochastic simulation with one individual per site, (an extension of an Eden model variant including different genotypes, see also Fig. 2a of Ref. [5]). On a hexagonal lattice, each lattice site can be occupied by one individual with a given genotype, represented by the lattice site being assigned a given color. The individual (or the lattice site) can reproduce by converting a neighboring, empty lattice site into a site of the same color. At a given time, a site with at least one empty neighboring site (essentially an individual at the front) is chosen at random (here: a green one) and randomly converts one of the empty neighboring sites (two possibilities, indicated by arrows). Time is updated by a random number drawn from an exponential distribution with the mean being the inverse of the number of sites with at least one empty neighboring site. The gray sites representing the obstacle cannot be occupied and are regarded as filled non-reproducing sites in the simulation.
(TIF)

S13 Fig Plaque growth on a heterogeneous bacterial lawn. **(A)** Bacteriophage inoculation on a heterogeneous lawn. After inoculation with a carrier fluid that evaporates quickly, a mixture of susceptible (yellow) and resistant (red) cells grows into micro-colonies representing an environment with a large number of obstacles to phage growth. Right before the colony was imaged, phage T7 was inoculated on the left part of the lawn, at the location indicated by the arrow. (The black gaps inside the lawn represent the absence of bacteria and are filled in in the course of the experiment.) **(B)** Bacteriophage spread on a heterogeneous lawn. After approximately 12 hours, the plaque (dark region due to lysis of bacteria) has extended through about half of the colony, almost exclusively affecting the susceptible part of the lawn. The regions of resistant bacteria cause transient perturbations in the front of the phage population, as seen in the close-up of the region indicated with an arrow. See S8 Video for all frames, Materials and Methods for more information, and S9 Video for a control experiment without resistant cells.
(TIF)

S14 FigShape of the population profile far away from the obstacle for the reaction-diffusion model.Profile is determined at the boundary of the lattice for the numerical solution displayed in S2 Video after the front has encountered the obstacle. The black line indicates the approximation *u*(*z*) ≈ (1 + *e* ^*z*/*c*^)^−1^ + 1/4 ⋅ *e* ^*z*/*c*^(1 + *e* ^*z*/*c*^)^−2^ ln(4*e* ^*z*/*c*^(1 + *e* ^*z*/*c*^)^−2^) with $z=y^{\prime }\sqrt{{\hat{k}}_{\text{eff}}/{\hat{D}}_{\text{eff}}}$, where *y*′ is the position in the comoving frame, ${\hat{k}}_{\text{eff}}$ and ${\hat{D}}_{\text{eff}}$ are the growth rate and diffusion coefficient as specified in Materials and Methods, and *c* = 2 is the dimensionless front speed; see Ref. [43] for more details.(TIF)
