# Supplementary material for: How Obstacles Perturb Population Fronts and Alter Their Genetic Structure
Source: PLoS Comput Biol. 2015 Dec 22;11(12):e1004615. doi: 10.1371/journal.pcbi.1004615 (PMC4690605; doi:10.1371/journal.pcbi.1004615)
Supplement: S1 Appendix — (PDF) [file pcbi.1004615.s010.pdf]

S1 Appendix: The Constant Speed Model

How obstacles perturb population fronts and alter their genetic structure

Wolfram Möbius, Andrew W. Murray, and David R. Nelson

Contents

1 Constant speed model for obstacles of different shape 1
1.1 Rhombus-shaped obstacle 1
1.2 Other convex obstacles with bilateral symmetry 2
1.3 Circular obstacle 2
1.4 Elliptical obstacle 4
1.5 Tilted, convex, symmetric obstacles 5

2 Analogy to geometrical optics 5

3 Origin of the front's lag - limitations of the constant speed model 6

4 References 7

1 Constant speed model for obstacles of different shape

1.1 Rhombus-shaped obstacle

Assume a rhombus with width $2w$ and height $2h$ with its width parallel to a population front encountering this obstacle as sketched in Fig. 2H. The position of the population front at a given time is characterized by the distance d traveled normal to the front (Fig. 2F). The constant speed model predicts that the front remains planar until the point of maximum width is reached (conveniently defined as $d = 0$, see main text and Fig. 2F). If the speed v of the front remains constant over the relevant time interval, we have $d = vt$. More generally, $d = \int_0^t v(t')\mathrm{d}t'$. Note that the prediction of the front shape is independent of $v(t)$, but we chose the term 'constant speed model' for clarity. The curved part of the front, in the shadow of the obstacle, is given by the arcs of two circles with radius d. A kink forms at $d = \sqrt{w^2 + h^2}$ and is characterized by opening angle ϕ (see S8 Fig for the analogous opening angle ϕ for a circular obstacle) and indent size Δ. Both are independent

of h (as is front shape, see Fig. 2H) and are given by:

$$\begin{aligned} \phi &= 2\arccos\left(\frac{w}{d}\right) \\ \Delta &= d\left(1-\sqrt{1-\frac{w^2}{d^2}}\right). \end{aligned} \tag{1}$$

For $d \gg w$ we obtain

$$\begin{aligned} \phi &\approx \pi - \frac{2w}{d} \\ \Delta &\approx \frac{w^2}{2d}. \end{aligned} \tag{2}$$

1.2 Other convex obstacles with bilateral symmetry

Aligned rhombus-shaped obstacles are simple in the sense that the constant speed model predicts initiation of two radial population waves simultaneously at $d = 0$, when the traveling wave passes the point of maximum width. This simplicity also holds true for rectangular obstacles (aligned parallel to the population front), where radial population waves are initiated when the front has just finished grazing the sides of the obstacle. For more complex obstacles, multiple radial population waves can be initiated. Let us first consider general convex obstacles with bilateral symmetry before quantifying the considerations for circular and elliptical obstacles below (see S8 Fig).

According to the constant speed model, the front of the population wave at distance d is determined by an ensemble of paths of length d all hugging the boundary before continuing tangentially. There is thus a successive initiation of circular population waves along the boundary of the obstacle. Hence, the tangents to the front and the boundary of the obstacle form a 90° angle after the front has passed the point of maximum width as shown in the left-most part of S8 Fig.

For long times (and large distances downstream from the obstacle) the front is completely determined by segments originating around the region of maximum width (S8 Fig). The front in this limit is thus only determined by the shape of the obstacle near the point of maximum width and ultimately only by the width of the obstacle $2w$. Hence, asymptotic results such as Eqs. 2 hold independent of the detailed obstacle shape.

1.3 Circular obstacle

The above considerations for convex shapes with bilateral symmetry are conveniently illustrated with a circular obstacle. (The more general case of elliptical obstacles will be treated later.) For a circular obstacle (radius r) it is convenient to use the polar angle θ to parametrize the boundary (S8 Fig). From the constant speed model sketched above, we find the coordinates for the right part of the front parametrically as function of θ at fixed

$d(t) = \int_0^t v(t')\mathrm{d}t$ as

$$\begin{aligned} x(\theta) &= r\cos\theta - (d - \theta r)\sin\theta \\ y(\theta) &= r\sin\theta + (d - \theta r)\cos\theta\,, \end{aligned} \tag{3}$$

where $x(\theta)$ and $y(\theta)$ are given relative to the center of the obstacle.

The results hold for all positive distances d relative to the midline of the circle parallel to the initial front, but the maximum value of $\theta = \hat{\theta}$ allowed in Eq. 3 depends on how far the wave has traveled:

$$\begin{aligned} 0 \le \theta \le \frac{d}{r} \qquad &\text{if} \qquad d \le \frac{\pi r}{2} \text{ (i.e., until the kink forms)} \\ 0 \le \theta \le \hat{\theta} \qquad &\text{if} \qquad d > \frac{\pi r}{2} \text{ (i.e., after the kink forms)} \end{aligned} \tag{4}$$

where $\hat{\theta}$ is determined by the condition $x(\hat{\theta}) = 0$:

$$\cot\hat{\theta} + \hat{\theta} = \frac{d}{r}\,. \tag{5}$$

The kink shape at its birth follows from expanding x and y around $\theta = \pi/2$ and eliminating θ. The cusp shape that marks the birth of the kink is given by

$$y(x) = r + \frac{1}{2}r\left(\frac{3x}{r}\right)^{2/3}\,. \tag{6}$$

Note that the initial opening angle ϕ vanishes in this case, i.e., $y(x)$ has an infinite slope.

The subsequent healing of the kink can be characterized similar to rhombus-shaped obstacles by the indent size Δ and opening angle ϕ:

$$\begin{aligned} \Delta &= d - y(\hat{\theta}) = d - r\sin\hat{\theta} - (d - \hat{\theta}r)\cos\hat{\theta} \\ \phi &= \pi - 2\hat{\theta}\,. \end{aligned} \tag{7}$$

The implicit Eq. 5 for $\hat{\theta}$ cannot be solved in closed form. However, one can consider the case of $d/r \gg 1$ (i.e., the front has already traveled several obstacle lengths downstream) which implies $\hat{\theta} \ll 1$. We make a polynomial ansatz for $\hat{\theta}$ as

$$\hat{\theta} = a_0 + a_1\frac{r}{d} + a_2\left(\frac{r}{d}\right)^2 + a_3\left(\frac{r}{d}\right)^3 + \ldots \tag{8}$$

Expanding the inverse of Eq. 5 and comparing coefficients on both sides of the equation we find

$$\hat{\theta} \approx \frac{r}{d} \quad \text{far from obstacle.} \tag{9}$$

Employing the polynomial representation of $\hat{\theta}$ and keeping the lowest terms depending on r/d we obtain for opening angle ϕ and indent size Δ:

$$\begin{aligned} \phi &\approx \pi - \frac{2r}{d} \quad \text{far from obstacle}, \\ \Delta &\approx \frac{r^2}{2d} \quad \text{far from obstacle}. \end{aligned} \tag{10}$$

Note that upon identifying r with w (half the maximum width of the obstacle), the opening angle and indent size of the kink agree with our earlier result, Eq. 2, for rhombus-shaped obstacles, consistent with universal large distance behavior for the kink.

1.4 Elliptical obstacle

The shape of the front encountering an obstacle shaped as an ellipse (oriented with one axis parallel to the unperturbed front) can be derived similarly. Assume here that the front encounters the ellipse parallel to the major axis of length $2a$ and with front direction parallel to the axis of length $2b$ with $a > b$. A parameter τ, $0 \leq \tau \leq 2\pi$, can be used to parametrize both the shape of the ellipse and the curve of the impinging front. Upon taking the center of the ellipse as the origin the boundary of the ellipse is given by $x_{\mathrm{el}}(\tau) = a\cos\tau$ and $y_{\mathrm{el}}(\tau) = b\sin\tau$. To compute the shape of the front we need the tangent to the ellipse and the arc length of the ellipse's boundary where the path is hugging the obstacle, both of which can be derived from the parametric form. In analogy to the circular case we find for the coordinates $x(\tau)$ and $y(\tau)$ of the right part of the front:

$$\begin{aligned} x(\tau) &= a\cos\tau - \left[d - aE\left(\frac{\pi}{2}, \sqrt{1-\frac{b^2}{a^2}}\right) + aE\left(\frac{\pi}{2} - \tau, \sqrt{1-\frac{b^2}{a^2}}\right)\right] \frac{a\sin\tau}{\sqrt{a^2\sin^2\tau + b^2\cos^2\tau}} \\ y(\tau) &= b\sin\tau + \left[d - aE\left(\frac{\pi}{2}, \sqrt{1-\frac{b^2}{a^2}}\right) + aE\left(\frac{\pi}{2} - \tau, \sqrt{1-\frac{b^2}{a^2}}\right)\right] \frac{b\cos\tau}{\sqrt{a^2\sin^2\tau + b^2\cos^2\tau}} \end{aligned} \tag{11}$$

where we used the incomplete elliptic integral of the second kind [1],

$$E(\phi, k) = \int_0^\phi \sqrt{1 - k^2\sin^2\rho}\,\mathrm{d}\rho \quad \text{with} \quad 0 < k^2 < 1, \tag{12}$$

to simplify the expression. Note that the parameter τ parametrizing the ellipse is similar but not identical to the polar angle $\theta = \tan^{-1}\left(\frac{b}{a}\tan\tau\right)$ measured from the ellipse center.

The kink forms when the wave has traveled a distance $d = aE(\pi/2, \sqrt{1 - b^2/a^2})$, along one quarter of the ellipse perimeter. Before the kink forms, the upper limit of τ is given by the condition that the expression in square brackets in Eqs. 11 is larger or equal to 0 while after formation of the kink the upper limit of τ is given by $x(\tau) \geq 0$.

To determine the shape of the kink at the moment it forms as a cusp one can set $d = aE(\pi/2, \sqrt{1 - b^2/a^2})$ and $\tau = \pi/2 - \epsilon$, expand for small ϵ, and express y as a function

of x resulting in:

$$y(x) = b + \frac{1}{2}\frac{(3a)^{2/3}}{b^{1/3}} x^{2/3}. \tag{13}$$

Again, note the vanishing opening angle and the characteristic power law $y(x) - b \sim x^{2/3}$.

1.5 Tilted, convex, symmetric obstacles

So far, only obstacles with bilateral symmetry relative to the front direction were considered. How do deviations in alignment affect the kink trajectory? To answer this question, we can use the constant speed model as shown in S9 Fig. Let us consider a thin flat obstacle with projected length $2L$ (onto the incoming front) and a vertical offset by $2h$ (resulting in a tilting angle of $\arctan(h/L)$). The trajectory of the kink, $(x_\mathrm{k}, y_\mathrm{k})$, is given by the intersection of two circles, one with radius $d + h$ centered at $(-L, -h)$ and another with radius $d - h$ centered at (L, h), where d is the distance of the front traveled in y-direction relative to a line parallel to the unperturbed front passing through the center of the obstacle. The resulting kink trajectory is

$$y_\mathrm{k} = \frac{L^2 h^2 + x_\mathrm{k}^2 \left(h^2 - L^2\right)}{2 x_\mathrm{k} L h}. \tag{14}$$

As indicated in S9 Fig, for large distances beyond the obstacle, the kink position slowly approaches the y-axis bisecting the projected obstacle, along a hyperbolic curve.

2 Analogy to geometrical optics

The constant speed model has a strong analogy to geometrical optics. The blue lines in Fig. 2H which mark the path of an imaginary particle at the front can be found by minimizing the path back to the front, which is equivalent to *Fermat's principle* minimizing the integral over optical density to infer the light path between two points [2, 3].

While the analogy to Fermat's principle is intuitive from a mathematical perspective, especially for extending predictions to more complex environments, the use of *Huygens's principle* gives an intuitive explanation for the construction of the wave front (black lines in Fig. 2H). Each point along the front is the source for a radial wave. The envelope of all these waves determines the wave front at a later time which intuitively explains the circular shape of the front in the shade of the obstacle [2]. Note that, of course, there is no interference as described by the Huygens-Fresnel principle [2].

Based on these analogies we also predict that the influence of non-perfect obstacles, which support traveling population waves with smaller speed, can be described by such a minimization procedure. Specifically, we believe that Snell's law of refraction (which can be derived from either description of geometrical optics) holds for population waves traversing boundaries between habitats in which the populations propagate with different speed.

Last, as explained in the main text, the constant speed model only is a good description of front shape if the size of the obstacle L is much larger than the front width parameter ξ. The analogous condition for geometrical optics is $\lambda \ll L$, where λ is the wavelength.

3 Origin of the front's lag - limitations of the constant speed model

S7 Fig illustrates two mechanisms which lead to a lag of the front relative to the constant speed model prediction as outlined in the main text. First, diffusion of phage into the obstacle leads to a local reduction of population density close to the boundary and in consequence to a lagging part of the front (S7 Fig, panel A). This lagging region forms a boundary layer with a width of the order of the diffusion length $\xi = \sqrt{\hat{D}_{\text{eff}}/\hat{k}_{\text{eff}}}$. Unless the size of the obstacle L satisfies $L \gg \xi$, the boundary layer is significant and can lead to an apparent lag compared to the prediction of the model of constant speed. As $L/\xi \to \infty$, the boundary layer still exists, but is only a minor perturbation of the overall front shape. Although phage diffusion into the obstacle most likely plays a role in our experiments, note that experimentally observed fronts encounter the boundary at an angle more closely resembling 90 ° (Figs. 1D,3A) than the population front originating from the FKPP equation (Fig. 3C). Although we attribute this difference to the coarse-graining of our model and differences in front detection, other effects in addition to the boundary layer could contribute to the lag. Further insights might arise from repeating the experiments and numerics using an obstacle with a reflecting boundary.

A second limitation arises because the constant speed model for rhombuses predicts a sudden change in the curvature of the front close to the position of maximum width, right when the circular arc arises (S7 Fig, panel B). Large curvatures can influence front speed of a Fisher population wave. To understand this effect, consider the case of uniform curvature, i.e., a radially expanding population in two dimensions. In radial coordinates and for a radially symmetric population density $u(r)$ the FKPP equation reads: $\partial u(r,t)/\partial t = \hat{D}_{\text{eff}}\partial^2 u(r,t)/\partial r^2 + (\hat{D}_{\text{eff}}/r) \cdot \partial u(r,t)/\partial r + \hat{k}_{\text{eff}} u(r,t)\,(1 - u(r,t))$. The second term only contributes significantly for radii of order the characteristic length ξ, but in this case leads to a slow-down of the wave [4]. We therefore expect a contribution to a lag of the front whenever circular arcs with initially very small radius are generated. As illustrated in S7 Fig (panel B), for a rhombus this is the case at the point of maximum width. We expect the relative contribution of this effect to front shape to decrease with increase of the obstacle size while keeping obstacle shape constant: For a rhombus, the slow-down due to high curvature occurs when the front grazes the obstacle for small and large obstacles alike, but the overall perturbation of the front is larger for larger obstacles, diminishing the importance of the lag. For obstacles without sharp corners but smooth boundaries, such as the circles and ellipses considered above, the constant speed model also predicts the emergence of circular arcs with very small curvature, in these cases all along the boundary. We therefore also expect a lag due to large curvature of the population front close to the

obstacle's boundary. More work is needed, however, to quantify the effect of this continuous generation of arcs with large curvature along a smooth boundary and its dependence on the obstacle's size.

4 References

[1] Weisstein EW. Elliptic integral of the second kind – from mathworld.wolfram.com; 2014.

[2] Hecht E. Optics. Addison-Wesley; 2001.

[3] Landau LD, Lifshitz EM. The classical theory of fields. Butterworth Heinemann; 2000.

[4] Murray JD. Mathematical biology, I. An introduction. 3rd ed. Springer; 2002.
