# Supplementary material for: How Obstacles Perturb Population Fronts and Alter Their Genetic Structure
Source: PLoS Comput Biol. 2015 Dec 22;11(12):e1004615. doi: 10.1371/journal.pcbi.1004615 (PMC4690605; doi:10.1371/journal.pcbi.1004615)
Supplement: S1 Protocol — (PDF) [file pcbi.1004615.s011.pdf]

S1 Protocol: Printing the Bacterial Lawn

How obstacles perturb population fronts and alter their genetic structure

Wolfram Möbius, Andrew W. Murray, and David R. Nelson

Contents

1 Choice of printer 1

2 Customizing the CD tray 2

3 Making agar patches and plates 2

4 Mapping from pattern to cartridges 2

5 Filling and refilling cartridges 3

6 Printing bacteria onto agar patches 3

7 References 5

In short, bacteria are printed from custom-filled ink cartridges onto agar patches placed onto the CD (Compact Disk) tray of the Epson Artisan 50 printer. Agar patches are created with a supporting membrane underneath. Cartridges are filled with bacterial cells suspended in 42% glycerol and are used to print bacteria onto the agar patch from a CMYK TIF image prepared with MATLAB. Additional cartridges with deionized water and 70% ethanol and original Epson cartridges with ink are used to flush the printhead, clean the printhead from bacterial solution and as a test of printhead function. S1 Fig displays the experimental setup and procedures. The following sections provide detailed protocols.

1 Choice of printer

For our experiments, an Epson Artisan 50 printer was used, since the protocol described here is based on the approach used in Ref. [1] (using an Epson R280 printer, a very similar model). In general, a printer is needed which (i) is able to print on CDs and therefore has

a CD tray on which agar patches can be placed during the printing process and (ii) uses piezoelectric material (instead of heat) to generate droplets (arguably more gentle to cells printed).

2 Customizing the CD tray

The CD tray of the Epson Artisan 50 printer serves as the supporting substrate on which the agar patches are located during the printing process (S1 Fig). Parts of a cut-up CD, glued into the CD tray, serve as spacers between the CD tray itself and components within the printer. With the position of CD parts and the agar patches carefully chosen, it is possible to minimize contact between the agar patch and the parts inside the printer which move and fixate the CD tray. Note that it might be necessary to cover openings in the CD tray which potentially serve to detect the absence of a CD by the printer driver. Preparation of several trays as support for the agar patches enables quick processing of replicate experiments. Trays were cleaned with detergent, rinsed with water and rubbed with ethanol to minimize contaminations.

3 Making agar patches and plates

1. Prepare warm molten agar: liquid 2xYT medium with 20 g/l agar and antibiotics as needed.
2. Produce agar patches: Pipette 10 ml of medium into standard plates (diameter 8.5 cm), place a $2 \times 3.5\,\mathrm{cm}^2$ piece of nitrocellulose membrane (Millipore, 0.8 μm AAWP) onto the solidified agar. Pipette 5 ml on top and distribute as well as possible.
3. Supporting plates: Pour 40 ml of medium into square plates (9 cm $\times$ 9 cm).
4. Keep plates in the dark at room temperature for two nights, then refrigerate if not used immediately.

4 Mapping from pattern to cartridges

When using a consumer inkjet printer to print a customized liquid, one challenge is to ensure the best possible mapping between colors used in the pattern and the cartridges used to print the pattern. One approach is to print one component at a time to avoid depositing liquid of the wrong type by the printer driver. For example, in an attempt to print a rich yellow, the printer driver might add some liquid from the black cartridge. We did not follow the approach of printing from one cartridge at a time [1] because it vastly slows down the printing protocol and small numbers of resistant bacteria in a region of predominantly susceptible bacteria do not influence the experiment and vice versa. As a practical matter, we found it easiest to design a template as a CMYK TIF image in MATLAB, which was printed using IrfanView and the Epson printer driver using Windows 7.

The Epson Artisan 50 printer prints with six cartridges containing yellow, black, cyan, magenta, light cyan, and light magenta inks. The yellow and black channels and specific driver options were heuristically chosen to minimize ambiguities in the assignment of colors to cartridges: ‘CD/DVD’, ‘Ultra Premium Photo Paper Glossy’, ‘Photo’, ‘A4 (210 x 297 mm)’, ‘Borders’, ‘Portrait’, ‘Fix Red-Eye’ unchecked, ‘High Speed’ unchecked, ‘Edge Smoothing’ unchecked, ‘Print Preview’ unchecked, ‘Black/Grayscale’ unchecked, ‘Color Management’ : ‘ICM’ & ‘Off (No Color Adjustment)’. Margins were adjusted to ensure the pattern was printed where the agar patches were placed, which can easily be tested by printing ink on paper mimicking the agar patches.

In the course of developing the printing assay the software package Gutenprint, providing an alternate printer driver, was also used to print patterns. The settings Gutenprint provides can be used for a non-ambiguous mapping, but this approach was not followed up in the course of this work.

5 Filling and refilling cartridges

- Refillable cartridge set T0781-T0786 from InkjetMall (East Topsham, VT) was used for our experiments. Unfortunately, this product is no longer sold, but any refillable cartridge set compatible with the printer of choice should work for the protocol outlined.
- Cartridges were filled deionized water, 70% ethanol and bacterial solution as described by the manufacturer. Note that filling the cartridges for the first time might differ from the procedure used to refill the cartridges.
- Cartridges with bacteria were used only once, cartridges with deionized water or ethanol were refilled when necessary and if contaminations could be ruled out.
- The protocol outlined below requires significantly more yellow and black cartridges than cartridges of any other color. Costs can be reduced by swapping the chips between cartridges (if possible), which allows one to use cartridges originally meant for other colors than yellow or black to be used with bacterial solution.

6 Printing bacteria onto agar patches

The protocol below reflects the printing assay and cartridges used in this paper, but can easily be adjusted to print two arbitrary bacterial strains.

1. The day before: Make overnight culture of eWM43 and eWM44 from single colony in 17 ml 2xYT with 100 μg/ml ampicillin.
2. Spin down 5 ml of eWM43 and 15 ml of eWM44 culture. Resuspend cells in 15 ml of 42% glycerol by vortexing.
3. Fill empty cartridges with bacterial solution using a syringe by creating a vacuum inside the cartridge which is replaced by the bacterial solution. Remove air vent tabs

of cartridges afterwards.

4. With Epson cartridges in printer, perform nozzle check as provided by Epson software. Ensure all nozzles are working properly.
5. Replace cartridges by custom cartridges filled with deionized water. Perform ‘head cleaning’ twice to flush printhead. Perform nozzle check to ensure no ink is left in the printhead.
6. Print one pattern using software IrfanView on agar patches placed on CD tray with printer options as specified above. Transfer agar patches to agar-filled plate and incubate at 37 °C. Here, deionized water is printed and incubation of these patches thus serves to test for contaminations.
7. Replace yellow cartridge filled with deionized water by yellow cartridge filled with bacterial solution (eWM43, *E. coli* susceptible to bacteriophage T7 infection). Perform ‘head cleaning’ twice to flush bacterial solution into the printhead.
8. Print one pattern using the same settings as before and transfer agar patches to agar-filled plate. These patches serve as an estimate of how many susceptible cells are printed within the obstacle. After transfer to an agar-filled plate and incubation at 37 °C even single cells will occur as colonies which are easily detectable under a microscope. Note, however, that this test only concerns the printer driver and does not consider that printing properties can change when the the black cartridge filled with deionized water is replaced by a black cartridge filled with bacterial solution (next step).
9. Replace black cartridge filled with deionized water by black cartridge filled with bacterial solution (eWM44, *E. coli* resistant to bacteriophage T7). Perform ‘head cleaning’ twice to flush bacterial solution into the printhead.
10. Print different patterns (with two obstacle shapes each) onto patches and transfer agar patches to agar-filled plate. Incubate at 37 °C before adding phage and imaging as explained in Materials & Methods.
11. Insert cartridges with 70% ethanol into printer. Perform ‘head cleaning’ twice to clean printhead.
12. Insert original Epson cartridges into printer. Perform ‘head cleaning’ twice to flush original Epson ink into printhead.

During the printing process, two types of errors can occur and should be corrected as follows:

- Since cartridges that are filled with deionized water or ethanol are refilled and reused, they can be reported as empty by the printer even though sufficient amount of deionized water or ethanol is still left in the cartridge. Furthermore, cartridges occasionally are not recognized by the printer. If such errors occur, the cartridge is reinserted, the protocol adjusted appropriately (e.g., by performing ‘head cleaning’), and the experiment continued.
- Parts of the printer can have reached their end of life, for example the ink pad storing the ink used, e.g., in head cleaning, can be full. In principle, the ink pad can

be replaced manually and the counter can be reset for the Epson Artisan 50. Since this did not work reliably, however, we used a new printer.

7 References

[1] Cohen DJ, Morfino RC, Maharbiz MM. A modified consumer inkjet for spatiotemporal control of gene expression. PLoS ONE. 2009; 4:e7086.
