# Supplementary figures and images for: How Obstacles Perturb Population Fronts and Alter Their Genetic Structure
Source: PLoS Comput Biol. 2015 Dec 22;11(12):e1004615. doi: 10.1371/journal.pcbi.1004615 (PMC4690605; doi:10.1371/journal.pcbi.1004615)

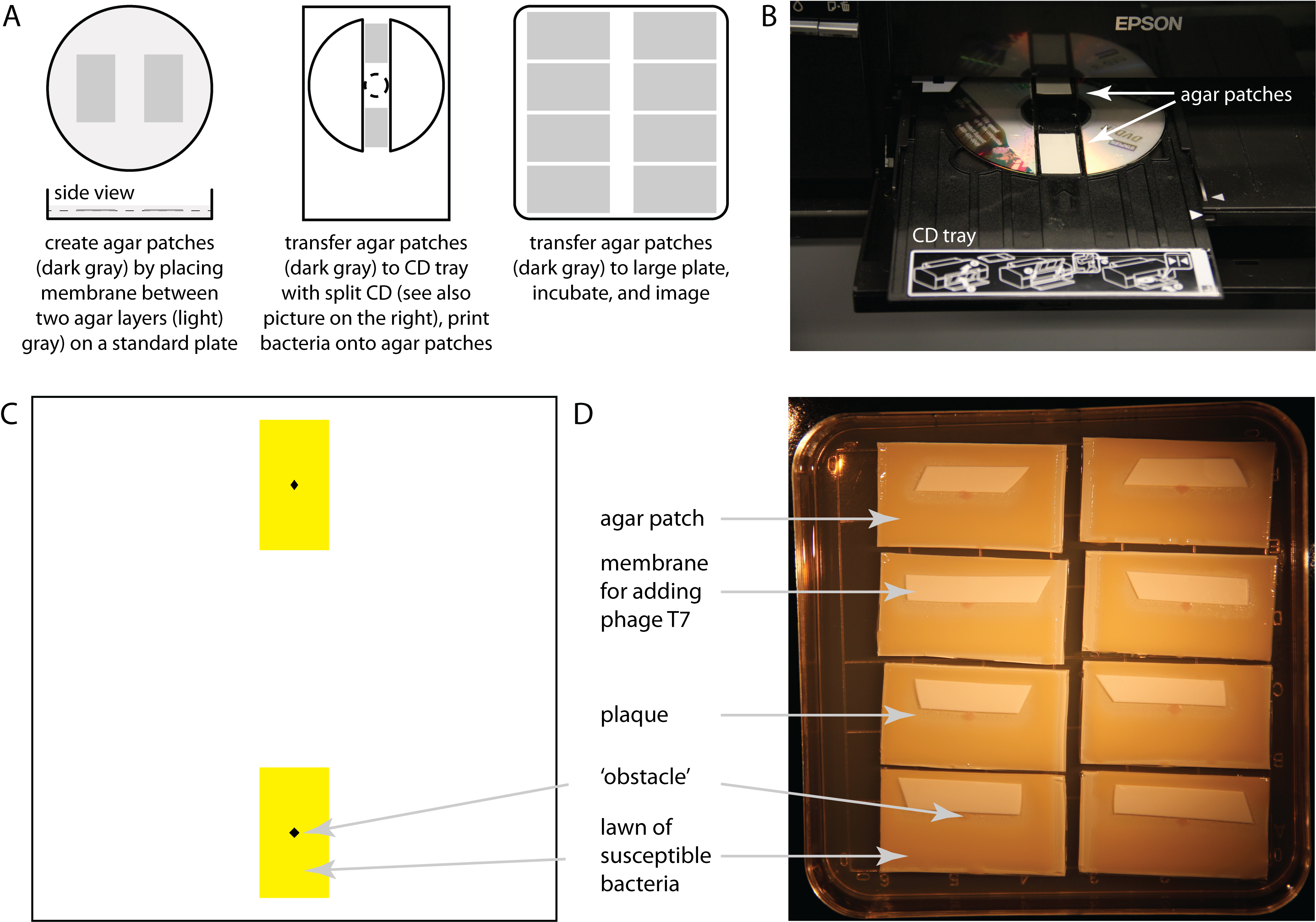

Supplement: S1 Fig — (A) First, agar patches are created by placing a membrane (cut to 3.5 × 2 mm2) between two layers of agar. The membrane with the top layer of agar is cut out and placed onto the printer’s CD tray. A split CD is used to define position and ensure proper functioning of the printer. After printing of the bacterial solution, the agar patches are transferred to a larger agar plate which then is incubated. (B) Photograph of the CD tray with two agar patches loaded into the printer (Epson Artisan 50). (C) Image of the pattern used to print bacteria onto the agar patch. Susceptible bacteria are found in the yellow cartridge, resistant bacteria in the black cartridge which after printing leads to the pattern described in Fig 1C. (D) Picture of the plate with experiments on plaque growth around single obstacles, after incubation and imaging. (TIF) [file pcbi.1004615.s012.tif]

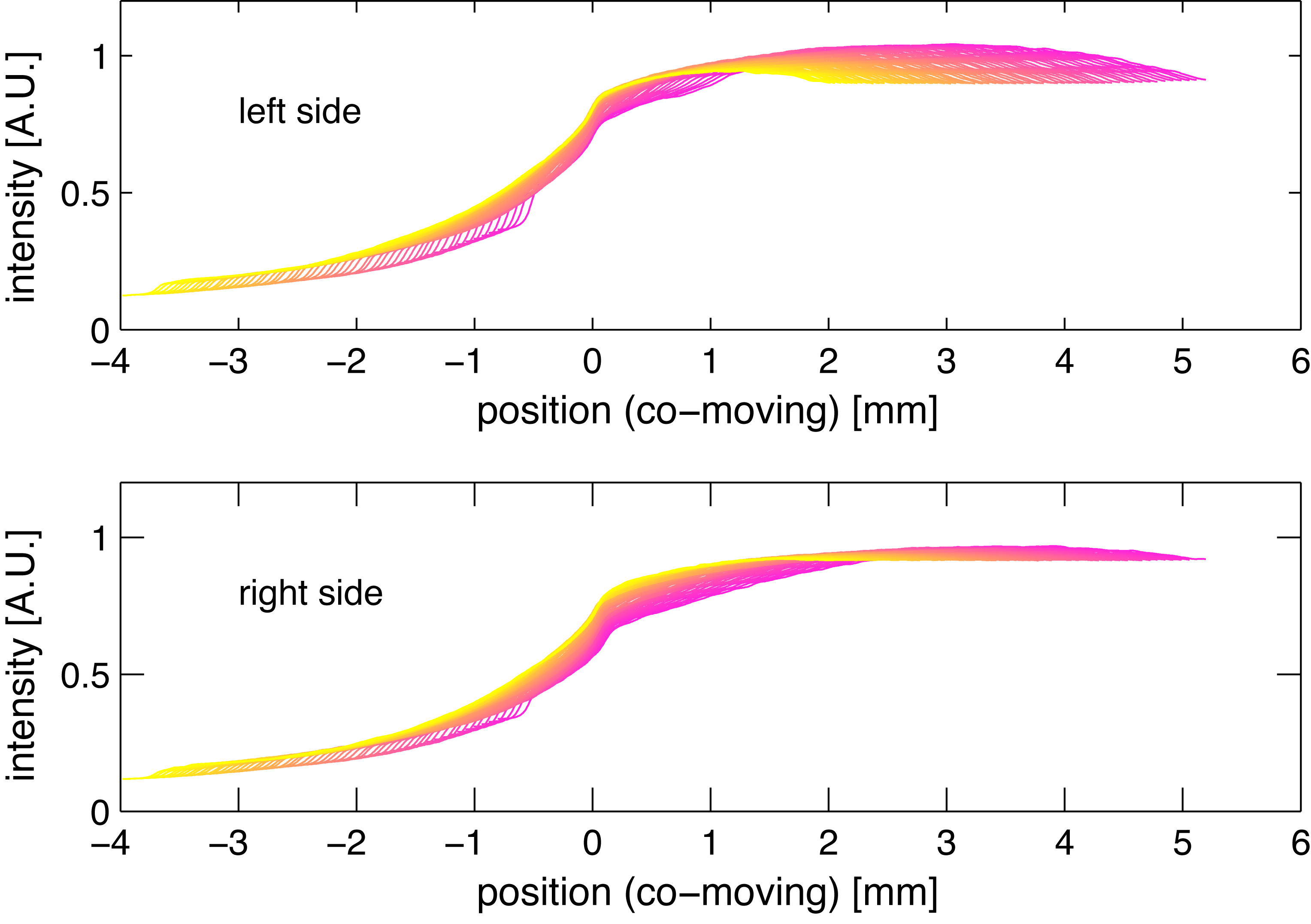

Supplement: S2 Fig — Different colors represent different time points where yellow corresponds to late times after the start of the experiment. Different front profiles do not collapse onto each other perfectly (and do so less well in other experiments), but the small differences are not expected to influence our results on front shape since algorithmic front detection was checked manually. (TIF) [file pcbi.1004615.s013.tif]

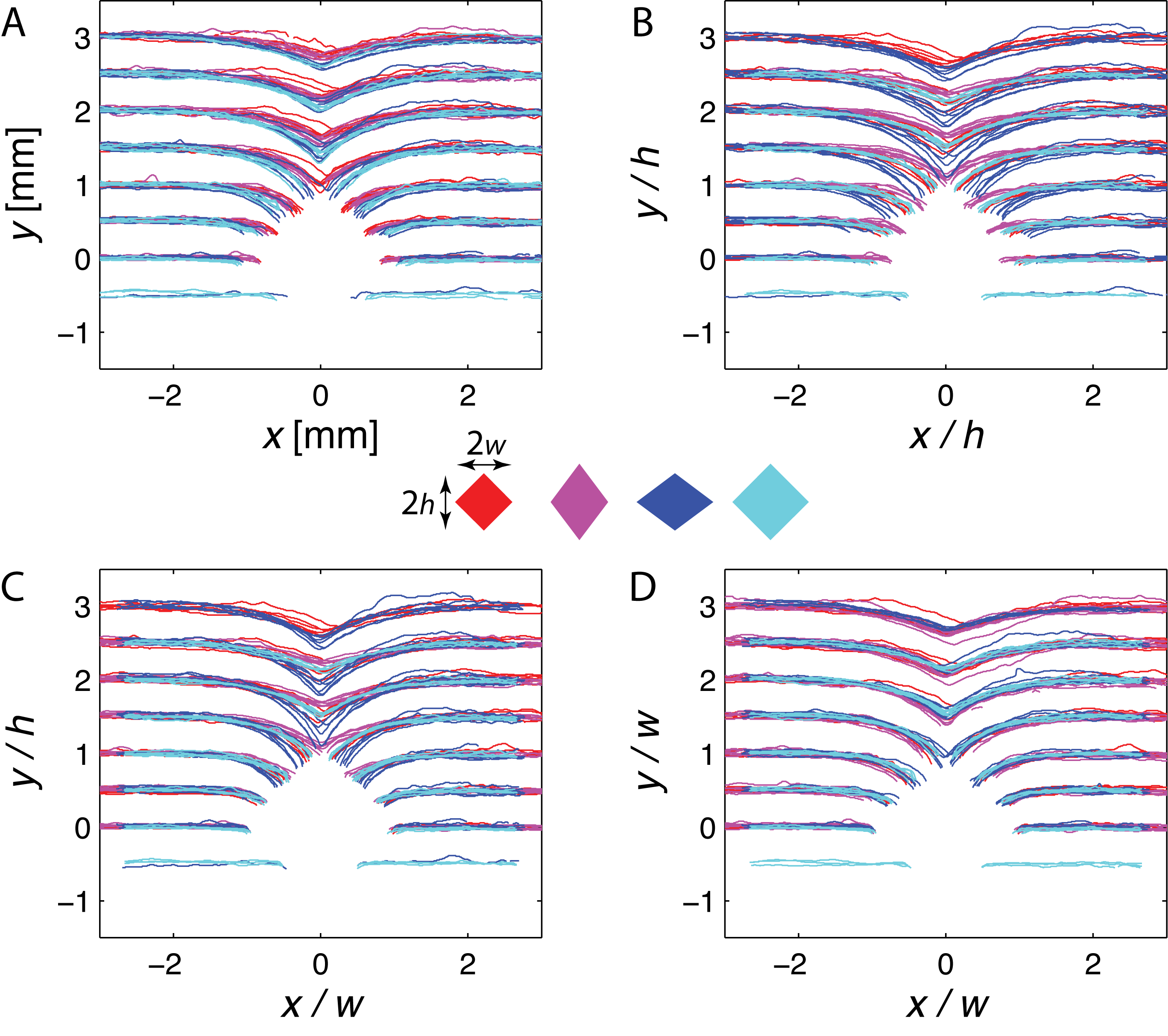

Supplement: S3 Fig — Axes are not scaled or scaled by half the obstacle width, w, or half the obstacle height, h, as indicated (see also Fig 3A). Collapse is best when all lengths are measured in units of w (panel D, Fig 3A) as predicted by model of constant speed. The collapse of front shapes for obstacles of same width (but not same height) in panel (A) illustrates that width, not height, of the obstacle determines front shape for rhombus-shaped obstacles. (TIF) [file pcbi.1004615.s014.tif]

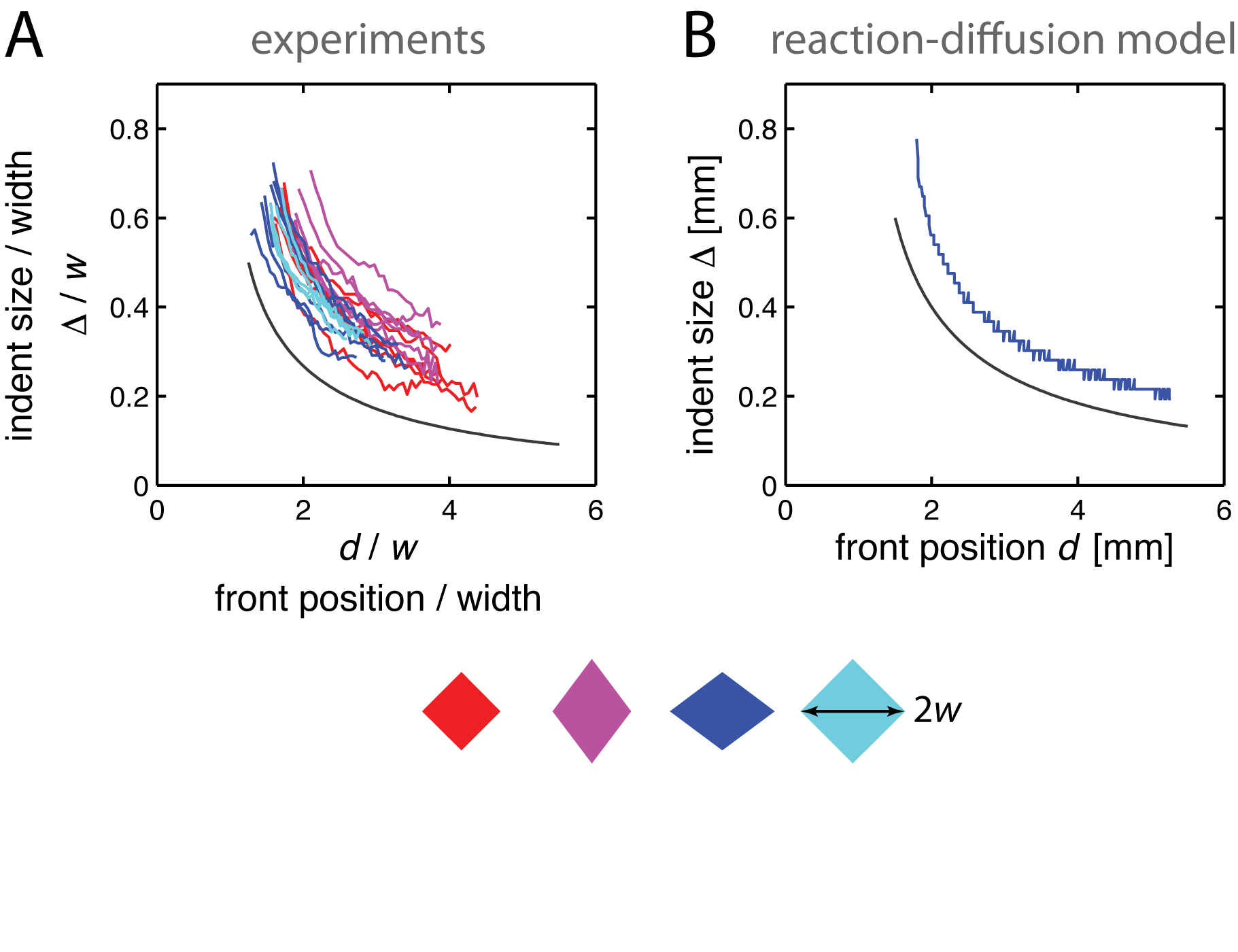

Supplement: S4 Fig — The experimental data are scaled by half the obstacle width, w, compare Fig 3B and 3D of the main text. (TIF) [file pcbi.1004615.s015.tif]

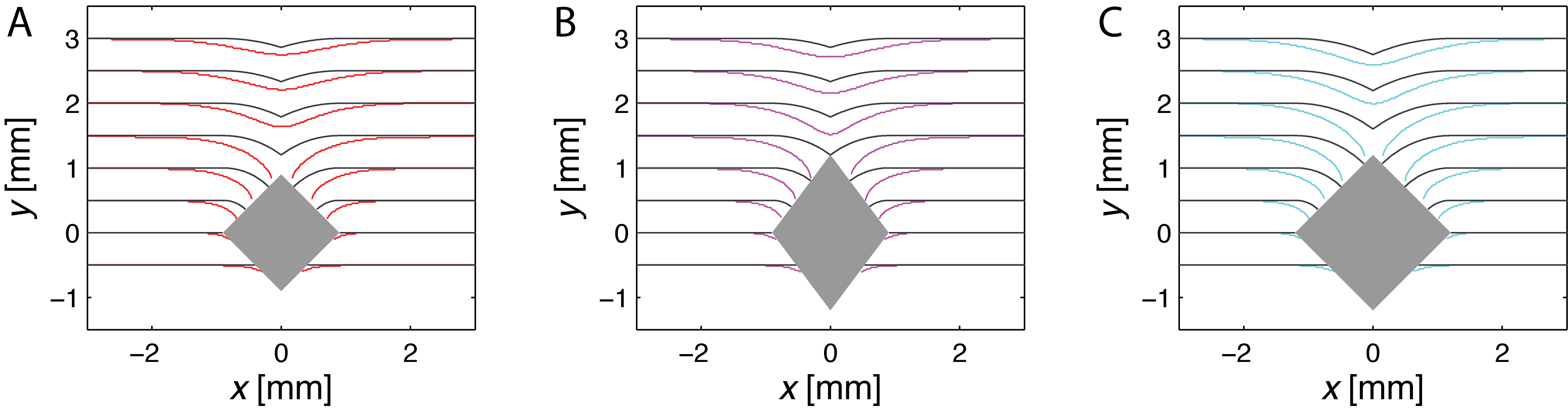

Supplement: S5 Fig — In all cases, the front in the reaction-diffusion model lags behind the front in the model of constant speed. (TIF) [file pcbi.1004615.s016.tif]

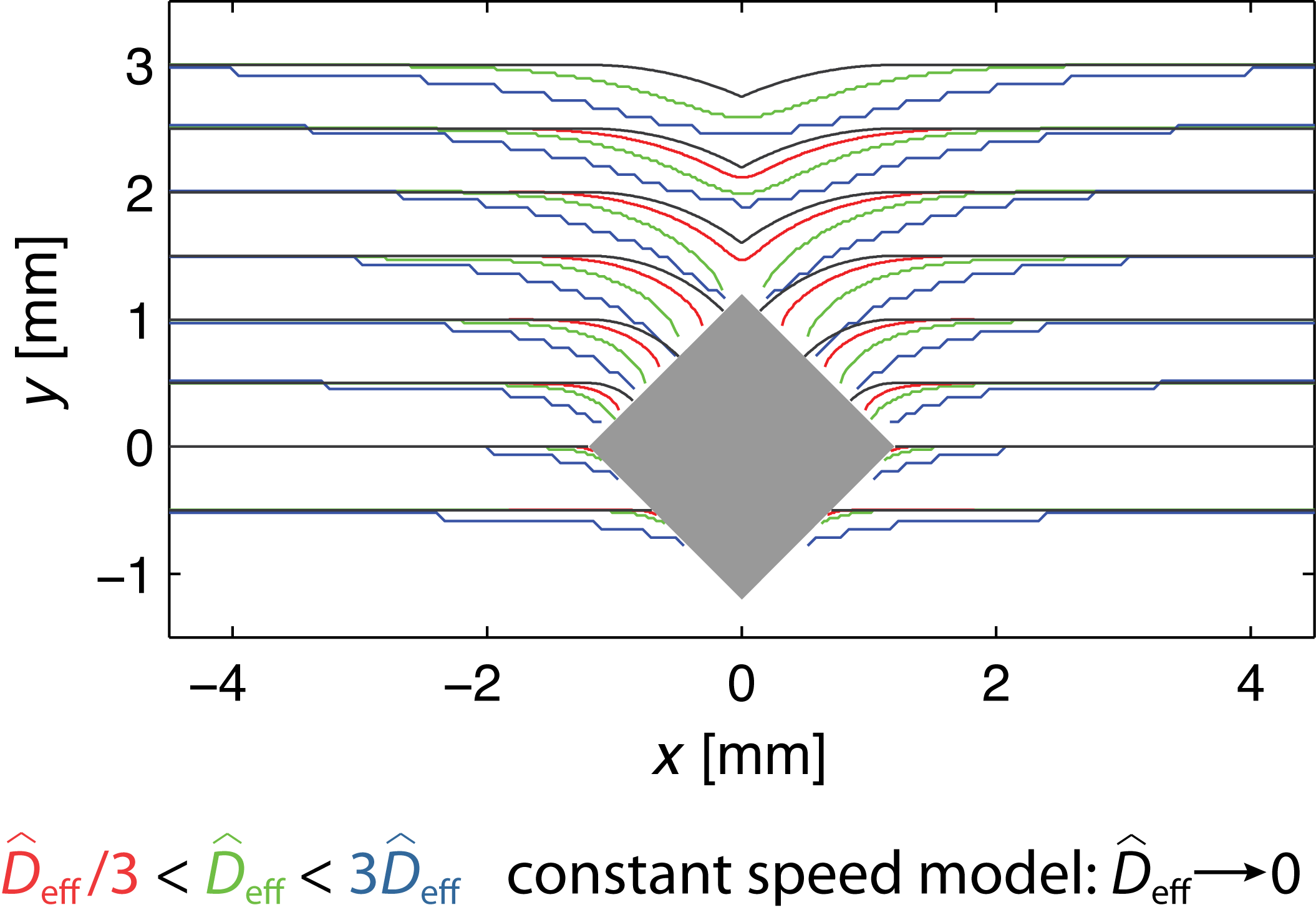

Supplement: S6 Fig — (Choice of diffusion coefficient determines effective growth rate as explained in the main text.) Green: D^eff=0.0144mm2/h, red: D^eff→D^eff/3, blue: D^eff→3D^eff, see Materials and Methods for justification of the choices for D^eff. In all cases, the front in the reaction-diffusion model lags behind the front predicted by the model of constant speed. The reduction of the lag with decreasing D^eff is consistent with a decrease in ξ=D^eff/k^eff which characterizes the limit of the model of constant front speed. (TIF) [file pcbi.1004615.s017.tif]

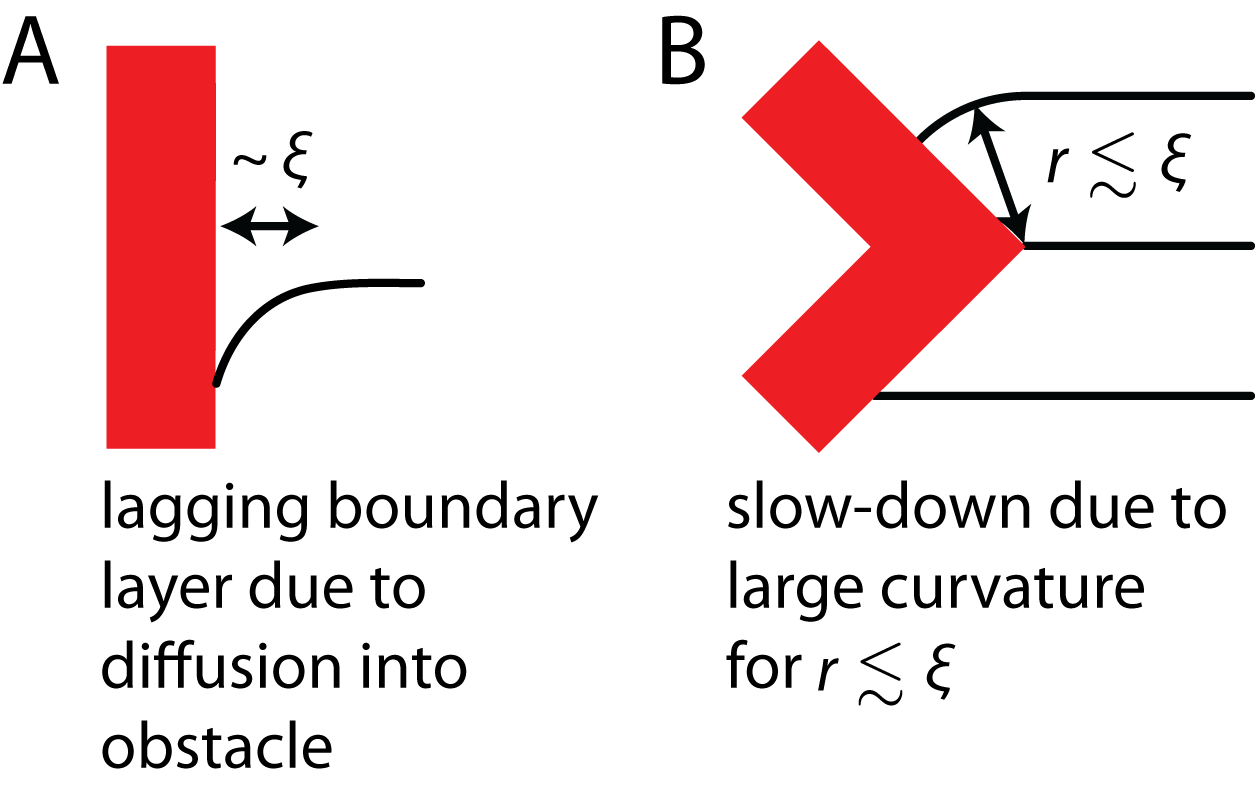

Supplement: S7 Fig — (A) Diffusion of the reproducing phage into the obstacle leads to a lagging front due to a boundary layer of width ∼ξ=D^eff/k^eff, where D^eff is the diffusion coefficient inside and outside the obstacle and k^eff the growth rate outside the obstacle. (B) A (rapid) change in the slope of an obstacle boundary can induce a lag while the radius r of the circular segments of the population front is smaller than the characteristic length scale ξ, r≲ξ. See text and S1 Appendix for details. (TIF) [file pcbi.1004615.s018.tif]

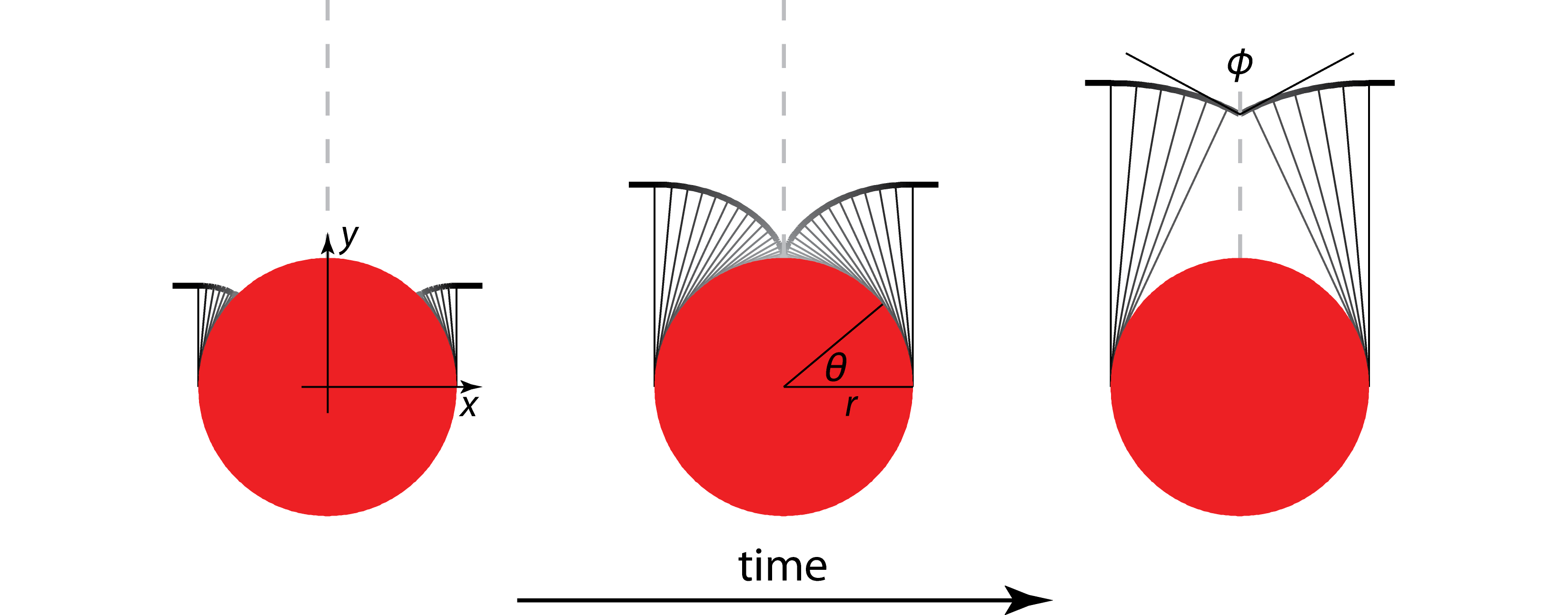

Supplement: S8 Fig — At any given time, the front in the shadow of the obstacle is composed of infinitely many circular segments with centers at the boundary of the circle. Since the upper boundary of the obstacle is parallel to the original front, the opening angle ϕ of the kink vanishes at the moment the kink forms, resulting in two locally parallel population fronts. (The kink therefore is a cusp in this case.) As the kink heals, circular inflation still occurs locally, but the arc length of the perturbed front is reduced. At large times during the healing phase fewer and fewer circular segments contribute to the front: Finally, only those with centers close to the point of maximum width are relevant. In this long time, large distance limit, the detailed shape of the obstacle drops out. In addition, the coordinate system and the parameter θ used to describe the circle are indicated. (TIF) [file pcbi.1004615.s019.tif]

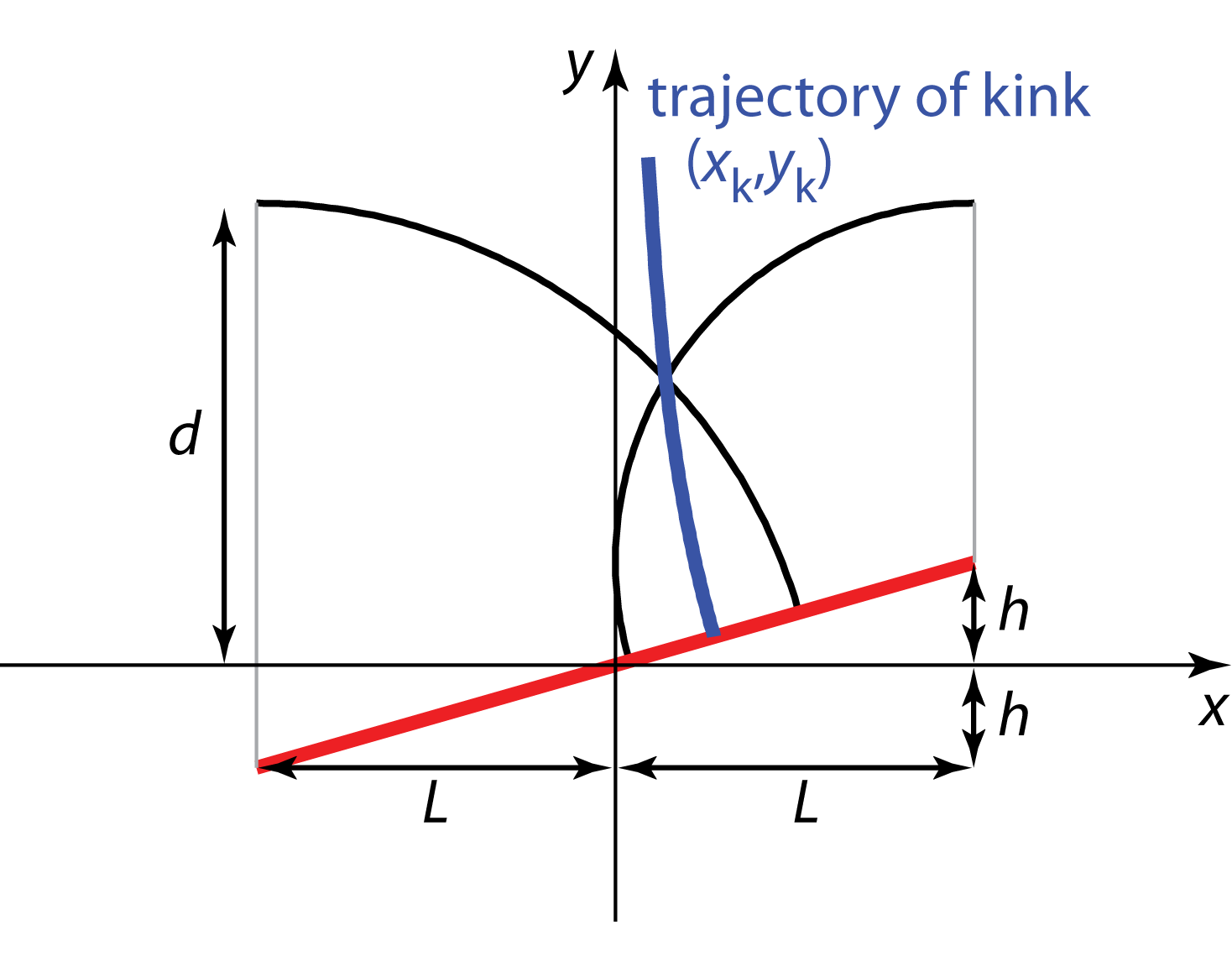

Supplement: S9 Fig — The projected width of the obstacle is 2L, the obstacle is tilted by an angle arctan(h/L). Black segments of circles centered on the edges of the obstacle indicate front shape at a given point during the healing process. The kink forms off-center, but its tip approaches a line normal to the unperturbed population front that bisects the projected obstacle (curved blue line). See S1 Appendix for details. (TIF) [file pcbi.1004615.s020.tif]

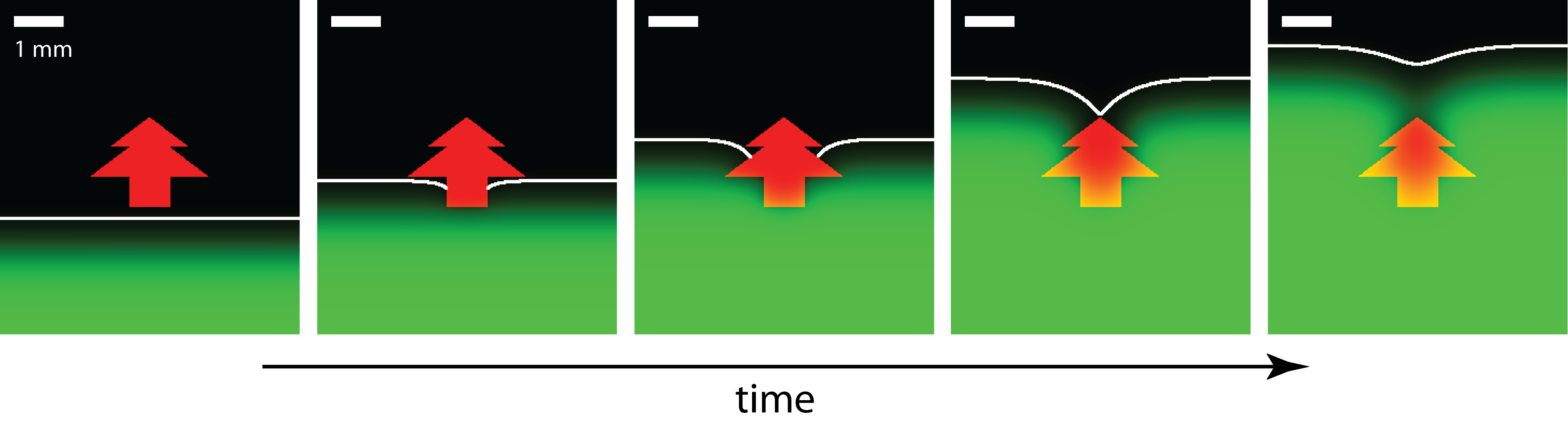

Supplement: S10 Fig — The region of no growth is indicated in red. Population density is indicated in green, the inferred front of the traveling population wave is marked white. See S3 Video for all frames and compare to Fig 3E for a rhombus-shaped obstacle. (TIF) [file pcbi.1004615.s021.tif]

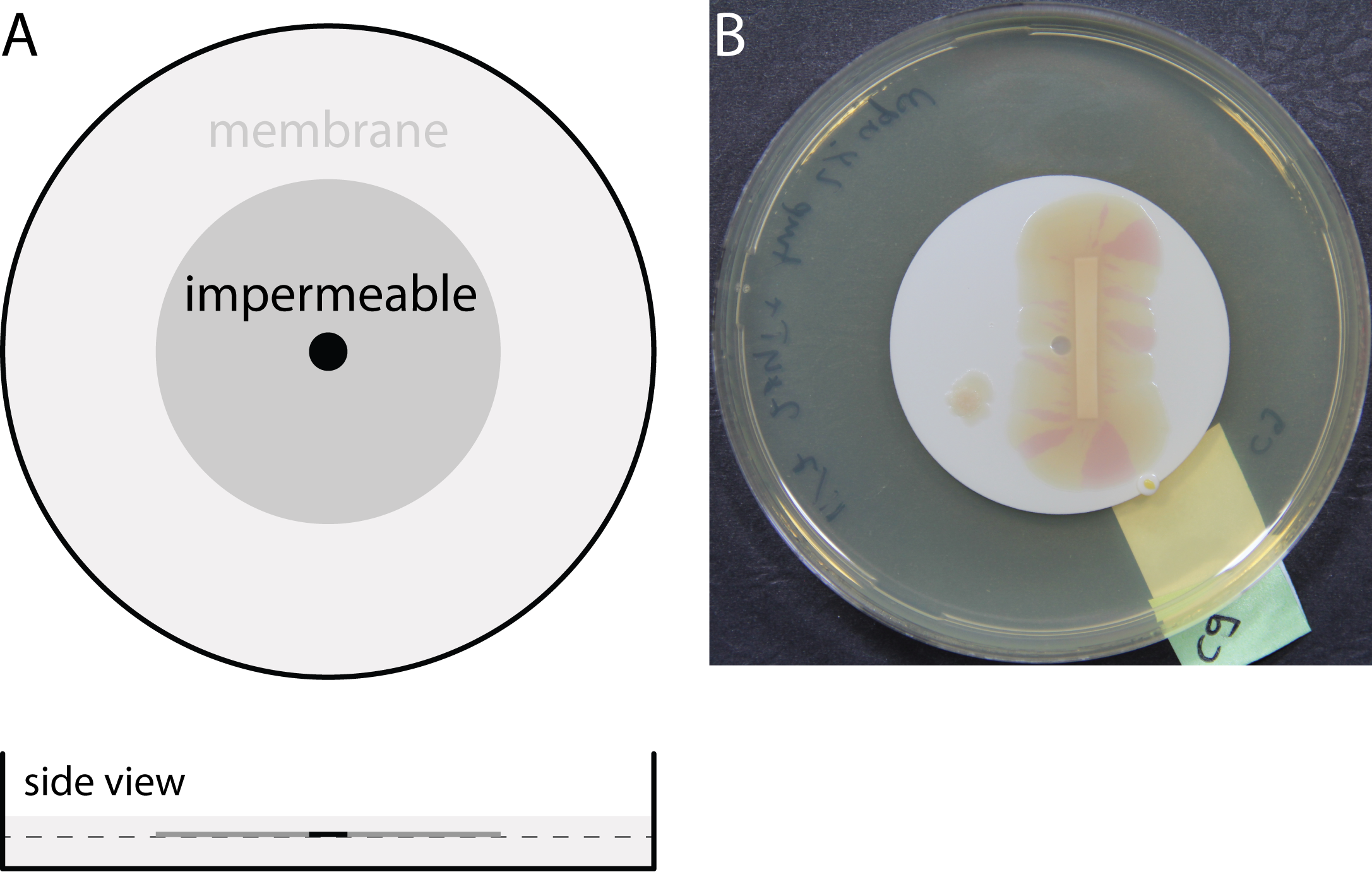

Supplement: S11 Fig — (A) A part of a membrane is first made impermeable (see Materials & Methods). The membrane is then placed on top of an agar layer and covered by a thin layer of agar. (B) Picture of plate after bacterial expansion took place. (TIF) [file pcbi.1004615.s022.tif]

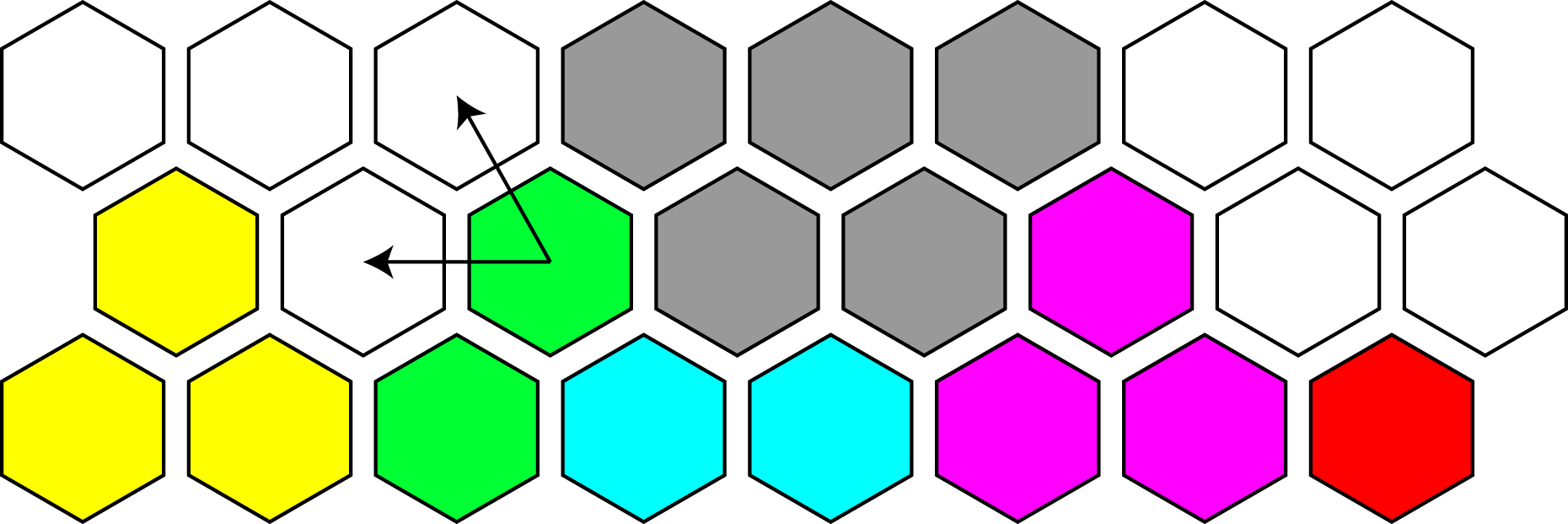

Supplement: S12 Fig — On a hexagonal lattice, each lattice site can be occupied by one individual with a given genotype, represented by the lattice site being assigned a given color. The individual (or the lattice site) can reproduce by converting a neighboring, empty lattice site into a site of the same color. At a given time, a site with at least one empty neighboring site (essentially an individual at the front) is chosen at random (here: a green one) and randomly converts one of the empty neighboring sites (two possibilities, indicated by arrows). Time is updated by a random number drawn from an exponential distribution with the mean being the inverse of the number of sites with at least one empty neighboring site. The gray sites representing the obstacle cannot be occupied and are regarded as filled non-reproducing sites in the simulation. (TIF) [file pcbi.1004615.s023.tif]

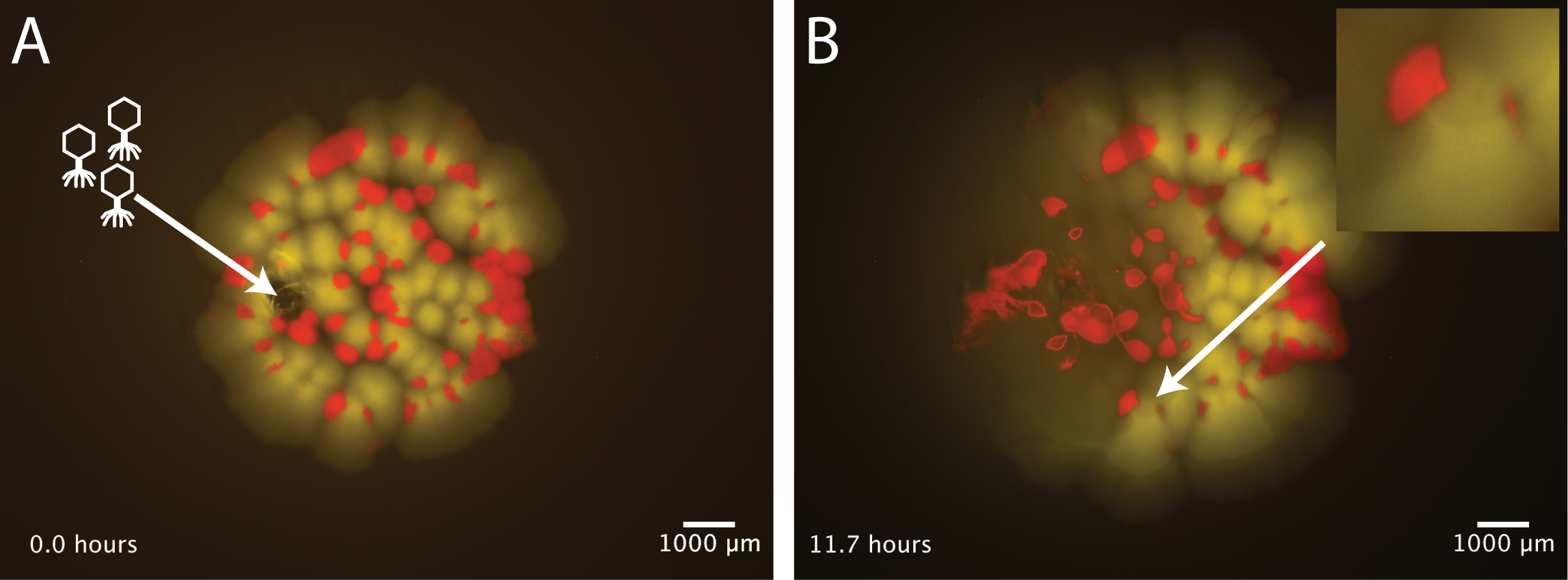

Supplement: S13 Fig — (A) Bacteriophage inoculation on a heterogeneous lawn. After inoculation with a carrier fluid that evaporates quickly, a mixture of susceptible (yellow) and resistant (red) cells grows into micro-colonies representing an environment with a large number of obstacles to phage growth. Right before the colony was imaged, phage T7 was inoculated on the left part of the lawn, at the location indicated by the arrow. (The black gaps inside the lawn represent the absence of bacteria and are filled in in the course of the experiment.) (B) Bacteriophage spread on a heterogeneous lawn. After approximately 12 hours, the plaque (dark region due to lysis of bacteria) has extended through about half of the colony, almost exclusively affecting the susceptible part of the lawn. The regions of resistant bacteria cause transient perturbations in the front of the phage population, as seen in the close-up of the region indicated with an arrow. See S8 Video for all frames, Materials and Methods for more information, and S9 Video for a control experiment without resistant cells. (TIF) [file pcbi.1004615.s024.tif]

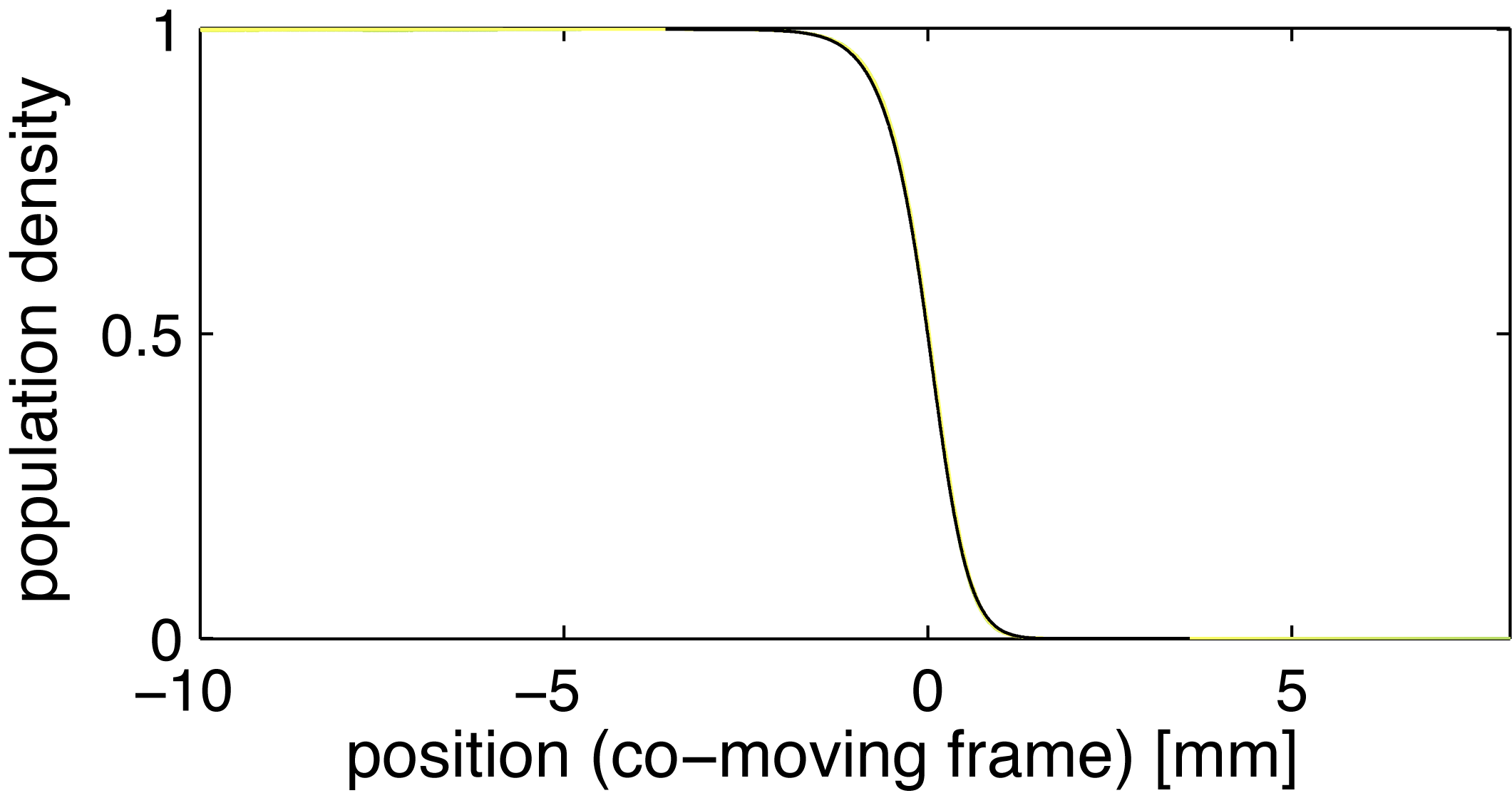

Supplement: S14 Fig — Profile is determined at the boundary of the lattice for the numerical solution displayed in S2 Video after the front has encountered the obstacle. The black line indicates the approximation u(z) ≈ (1 + e z/c)−1 + 1/4 ⋅ e z/c(1 + e z/c)−2 ln(4e z/c(1 + e z/c)−2) with z=y′k^eff/D^eff, where y′ is the position in the comoving frame, k^eff and D^eff are the growth rate and diffusion coefficient as specified in Materials and Methods, and c = 2 is the dimensionless front speed; see Ref. [43] for more details. (TIF) [file pcbi.1004615.s025.tif]
